# Cultural differences in explicit and implicit support provision and underlying motivations for self-esteem, closeness, and relational concerns

**DOI:** 10.3389/fpsyg.2023.1202729

**Published:** 2023-08-03

**Authors:** Rina Tanaka, Shaofeng Zheng, Keiko Ishii

**Affiliations:** ^1^Department of Cognitive and Psychological Sciences, Graduate School of Informatics, Nagoya University, Nagoya, Japan; ^2^Hitotsubashi Institute for Advanced Study, Hitotsubashi University, Kunitachi, Tokyo, Japan

**Keywords:** culture, social support provision, explicit vs. implicit social support, relational concerns, self-esteem

## Abstract

This research explores how culture influences the motivations underlying explicit (emotional and instrumental) and implicit (companionship and attentiveness) support provision. Two studies (*N* = 1,106) compared the responses of European Americans and Japanese individuals to a close other’s stressful event. The results showed that European Americans were more likely than Japanese to provide explicit support and more motivated to increase the close other’s self-esteem and feeling of closeness. Conversely, Japanese individuals were more likely to provide attentiveness support, motivated by concern for an entire group and a friend. These findings support the motivation as a mediator hypothesis. On the other hand, the culture as a moderator hypothesis applied to the association between concern for an entire group motivation and implicit support provision. Specifically, concern for an entire group motivation predicted companionship support provision only in Japanese, while it predicted attentiveness support provision mainly in European Americans.

## 1. Introduction

Many people experience daily stress that impacts their well-being and health, and social support is a highly effective means of coping with such stress (Seeman, 1996; Uchino, 2004). When social support meets the recipient’s needs and makes them feel understood and cared for, it is particularly beneficial (Cobb, 1976; Cohen and Wills, 1985). Although the importance of social support has been widely recognized, the ways in which social support transactions occur and the underlying motivations behind them differ across cultures, reflecting the influence of cultural values and norms for interpersonal relationships. European Americans tend to seek emotional comfort and instrumental aid more than East Asians (Taylor et al., 2004; Kim et al., 2008) and prioritize self-esteem motivations when seeking emotional and instrumental support (Ishii et al., 2017). On the other hand, emotional comfort obtained by perceiving a social relationship without disclosing the stressor is more beneficial for East Asians (Taylor et al., 2007), and Japanese people are more likely than European Americans to seek this implicit type of support by emphasizing relational concern motivations (Ishii et al., 2017). These cultural differences in seeking social support and underlying motivations are also reflected in the motivations for providing social support, with self-esteem motivations being positively associated with emotional and instrumental support provision in European Americans but not in Japanese (Chen et al., 2012). In this research, testing both Japanese and European Americans, we aim to investigate the manifestation of cultural values and norms for interpersonal relationships in the underlying motivations of individuals regarding social support provision. This aspect has not been fully understood previously. We examine how various forms of motivation influence both explicit and implicit types of social support provision across different cultures.

### 1.1. Culture and explicit and implicit social support

Cultural psychological research highlights cultural differences in the way individuals view themselves and their relationships with others (Triandis, 1989; Markus and Kitayama, 1991). The prevalent Western notion of an independent self, which involves perceiving oneself as separate from others and autonomous, emphasizes focusing on internal attributes such as traits and preferences, presenting oneself as unique, and influencing others. This form of self guides individuals toward maintaining and enhancing self-esteem and a sense of control. Conversely, the interdependent self, which is dominant in East Asian cultures, views the individual as connected to and interdependent with others. This form of self emphasizes focusing on relationships and communication with members of one’s in-group, adhering to shared norms, and fulfilling obligations and standards expected by the in-group. It guides individuals toward maintaining harmonious relationships and avoiding behaviors that disrupt group harmony.

Social support refers to expressions of caring and belonging within a social network that help individuals cope with stressors (Cobb, 1976). Although social support can be achieved in various ways, cultural psychology research has suggested that people’s preferences for a specific type of social support are influenced by their cultural norms in relationships. Emotional support, which provides comfort and reassurance, and instrumental support, which provides practical aid and advice, are two representative types of social support. Both types of support (called explicit support) are typically obtained by explicitly disclosing one’s needs and feelings. In Western culture, this kind of self-disclosure is considered important for achieving the shared value of independence, making it more normative than in East Asian culture (Taylor et al., 2007; Schug et al., 2010). Studies have found that European Americans were more likely than Asians and Asian Americans to seek explicit social support for coping with stressors (Taylor et al., 2004; Kim et al., 2006).

Furthermore, receiving explicit support from others (e.g., courage) during stressful times can help restore and maintain one’s positive self-image (i.e., self-esteem), which is more valued in Western culture than in East Asian culture. Research found that European Americans were more likely to seek explicit support for increasing self-esteem relative to Japanese individuals (Ishii et al., 2017). In addition to seeking explicit support, research conducted by Chen et al. (2012) found that, compared to Japanese individuals, European Americans were more likely to provide explicit social support, particularly emotional support, to their friends. This support was aimed at restoring their friends’ self-esteem in the face of stressors and strengthening the closeness of their relationships.

In contrast, East Asians, who emphasize relationship harmony, may prefer implicit social support more. Implicit social support is defined by the emotional comfort obtained from social networks without disclosing one’s stressors and needs (Taylor et al., 2007). Implicit social support can be provided by spending time with friends without discussing stressors or needs (i.e., companionship) and by monitoring friends who are stressed without offering tangible help (i.e., attentiveness; Chen et al., 2015). Relationships in Asian culture are relatively fixed and characterized by more obligation (Miller et al., 1990; Morling et al., 2002). Thus, East Asians are more cautious in discussing their personal needs to avoid burdening others. Research has demonstrated that Asians and Asian Americans are more likely than European Americans to be concerned about the negative impacts of disclosing stressful events on others in their social networks (i.e., relational concern; Taylor et al., 2004; Kim et al., 2006; Zheng et al., 2021). Moreover, research found that the motivation behind seeking implicit social support was more driven by relational concerns in Japanese than European Americans (Ishii et al., 2017). Chen et al. (2015) found that higher perceived closeness to a friend was associated with greater companionship and attentiveness, with this trend being stronger for Asian Americans compared to European Americans. However, it is unclear whether the utilization of implicit social support varies across cultures.

Taken together, the research reviewed above suggests that explicit social support is likely to be more prevalent and effective in Western culture, while implicit social support may be more normative in East Asian culture. Indeed, research has found that explicit social support was less effective in reducing stress responses and negative feelings in Asians and Asian Americans compared to European Americans (Taylor et al., 2007). In contrast, the perception of implicit social support was found to reduce stress and negative feelings more effectively in Asians and Asian Americans compared to European Americans (Taylor et al., 2007).

### 1.2. The remaining issues in culture and support provision: the roles of culture and motivation and the association between implicit social support and relational concerns

Considerable evidence has been accumulated on cultural influences on social support. However, relatively little research has examined how culture affects social support provision and the motivations behind it. Given these cultural differences in social support transactions, we aim to address two specific questions about the relationships between culture, motivation, and support provision in this research. The first question concerns the role of motivation for self-esteem in the relationship between culture and explicit social support. While cultural differences in explicit social support and motivation for self-esteem have been consistently found, it is unclear how motivation for self-esteem is related to culture and explicit social support provision. Chen et al. (2012) found a positive association between motivation for self-esteem and explicit support provision only in the US, whereas motivation for closeness predicted explicit support provision in the US and Japan. That is, culture moderated the association between the motivation for self-esteem and explicit support provision. On the other hand, given that Taylor et al. (2004) suggested that relational concerns play a mediating role in the relationship between culture and social support seeking, motivation for self-esteem may function as a mediator in the same way. In this case, explicit social support provision would increase as a function of motivation for self-esteem that differs cross-culturally. To elucidate relationships between culture, motivation, and social support provision, we tested two hypotheses: one based on culture as a moderator of the association between motivation and social support provision, and an alternative hypothesis based on motivation as a mediator of the effect of culture on social support provision.

Second, more importantly, while a cultural difference in the motivation for relational concerns in seeking implicit social support has been noted (Ishii et al., 2017), it has been unclear whether the provision of implicit social support varies across cultures and whether it is influenced by relational concerns across cultures. Specifically, the role that relational concerns motivation plays in the relationship between culture and implicit social support provision is also uncertain. Based on the culture as a moderator hypothesis, a positive association between motivation for relation concerns and implicit support provision would be observed, particularly in East Asians (e.g., Japanese). In contrast, based on the alternative hypothesis based on motivation as a mediator, implicit social support provision would increase as a function of relational concerns motivation, which differs across cultures.

To date, no research has addressed these two questions. This study aims to fill this gap in the literature by focusing on the strength of self-esteem motivation and relational concerns motivation and their role in the relationships between culture and explicit and implicit social support in Japanese and European Americans.

### 1.3. Two types of relational concerns in the context of support provision: concern for an entire group and concern for a friend

While research on cultural influences on social support provision and the motivations behind it is limited, it is expected that the findings will align with the existing literature on culture and social support seeking. However, the perspectives of support seekers and providers differ, which may be reflected in the aspects of relationships they are concerned about and the types of implicit support they provide. Support seekers are likely to consider how the other person and other members of their social group respond to their request for support, whereas support providers aim to help the person experiencing stressful events and problems while also considering the indirect effects of their support on surrounding in-group members. This emphasizes that both the needs of the person seeking support and the relationships among group members are important considerations in the context of social support provision.

The measure of relational concern, which has been exclusively used in previous research, was developed from the perspective of support seekers by Taylor et al. (2004). In their research, they categorized the reasons for avoiding social support seeking into five types: preserving social group harmony, the belief that disclosing the problem makes it worse, avoiding criticism, avoiding embarrassment, and a belief in self-reliance. After conducting a factor analysis, they found two latent variables. All five types of explanations loaded highly positively on the first factor (called relational concerns), which is characterized by a concern for maintaining relationships among in-group members and avoiding negative reputations. Conversely, self-reliance loaded highly positively, and criticism loaded highly negatively on the second factor (called independence concerns), which is characterized by the belief that individuals are expected to cope with problems on their own without concern for others’ views. Interestingly, cultural differences in social support seeking were explained by relational concerns, not independence concerns. However, due to the different perspectives between support seekers and providers, a concern corresponding to independence concerns, such as concern for the potential negative impacts on the support receiver, may not only exist but also play a more active role in implicit support provision.

We propose that there are two types of concerns that motivate people to provide implicit support. First, as support seekers, support providers may share a concern about discussing stressful experiences that would bother other group members besides the receivers, potentially disrupting the harmony of the entire group. Second, people may provide implicit social support simply to avoid causing harm to receivers. We refer to the former as “concern for an entire group” and the latter as “concern for a friend.” In this research, we investigate the strength of motivation for concern for an entire group and concern for a friend among Japanese and European Americans, as well as how these motivations are related to implicit social support provision.

### 1.4. Present research

**Figure 1:**
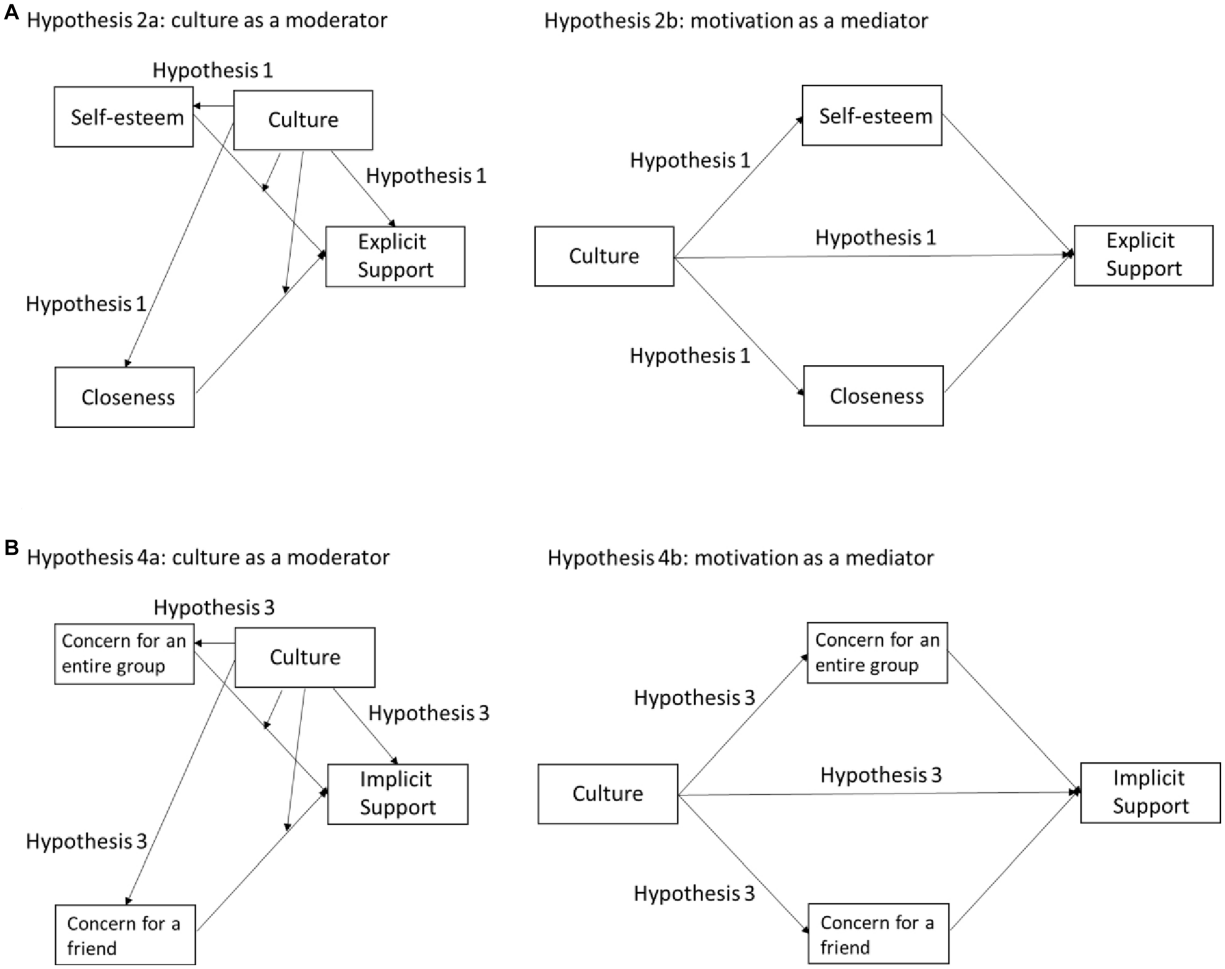
“Culture as a moderator” and “motivation as a mediator” models illustrating the associations among variables related to explicit support **(A)** and implicit support **(B)**.

We conducted two studies to explore the cultural influences on individuals’ motivations behind social support provision, with a focus on the provision of explicit and implicit social support and the motivations for self-esteem, closeness, concern for an entire group, and concern for a friend. Figure 1 presents the models that illustrate the associations among variables we hypothesize in this research. Study 1 examined the social support that Japanese and European American participants would provide to a close friend experiencing a hypothetical stressful event, and the motivations they would emphasize in making their decision. In Study 2, we directly asked Japanese and European American participants about the support they provided to a close friend who had recently experienced a stressful event and the motivations they emphasized. This research builds on the findings of Chen et al. (2012, 2015) and adds two new types of relational concern motivations and their relationships with two types of implicit social support provision (companionship and attentiveness). Building on the findings of Chen et al. (2012), we hypothesize that European Americans will provide more explicit support and prioritize self-esteem and closeness motivations to a greater extent than Japanese individuals (Hypothesis 1). Additionally, based on the culture as a moderator hypothesis suggested by Chen et al. (2012), greater self-esteem motivation would be associated with increased explicit social support provision in European Americans, while the trend would be weaker in Japanese (Hypothesis 2a). In contrast, based on the alternative hypothesis based on motivation as a mediator, self-esteem and closeness motivations would be positively associated with explicit social support provision, and the cultural differences in explicit social support provision would be explained by self-esteem and closeness motivations that are higher in European Americans than in Japanese (Hypothesis 2b).

Reflecting interdependence highly valued in Japanese, we expect that Japanese would provide more implicit social support and endorse more concern for an entire group motivation and concern for a friend motivation than European Americans (Hypothesis 3). Based on the culture as a moderator hypothesis, greater concern for an entire group motivation and greater concern for a friend motivation will be associated with increased implicit social support provision in Japanese, while the trend will be weaker in European Americans (Hypothesis 4a). In contrast, based on the alternative hypothesis based on motivation as a mediator, concern for an entire group motivation and concern for a friend motivation would be positively associated with implicit social support provision, and the cultural differences in implicit social support would be explained by concern for an entire group motivation and concern for a friend motivation that are higher in Japanese than in European Americans (Hypothesis 4b).

In addition, Chen et al. (2015) suggested that culturally appropriate ways of support provision are likely to occur in the case of close others (i.e., individuals with high-quality relationships). In both studies, we thus asked participants about the friend who was closest to them and had them rate how useful or disruptive the friend was in helping them achieve their goals. Although this measurement differs slightly from the one used to assess relationship quality, such as trust, intimacy, and satisfaction in Chen et al. (2015), we controlled for the ratings of the friend’s instrumentality in the following analyses. Moreover, the characteristics and perceptions of stressful events, which may vary across cultures, can influence the ratings for support provision and motivations. Therefore, we adopted the methods used in previous research (Kim et al., 2006; Ishii et al., 2017) to measure the characteristics and the perceptions of stressful events and addressed the potential influences. We also added two other types of motivation (self-improvement and low efficacy) for exploratory purposes and examined their strengths and associations with implicit social support across cultures, with the results presented in the Supplementary materials.

## 2. Study 1

### 2.1. Method

Both Studies 1 and 2 were reviewed and approved by the ethics committee at Nagoya University, Japan. All responses were kept confidential.

#### 2.1.1. Participants

We recruited 209 European Americans and 255 Japanese participants through online crowdsourcing marketplaces (Prolific for the European American participants and Lancers for the Japanese participants). According to a power analysis with G*Power 3.1, we needed at least 368 participants in total to detect a small effect size (*f^2^* = 0.03) with 80% power for an F-test (linear multiple regression: fixed model, R^2^ deviation from zero) when the significance level was set to 0.05. Thirty-one participants were excluded from the following analysis for the following reasons: Three Japanese participants failed to pass attention check questions, 25 Japanese participants did not complete more than half of the whole questionnaire, two Japanese participants did not allow us to analyze their data, and one European American participant did not report her or his age. Thus, the final sample size was 433 (208 European Americans [64.9% female, *M*_age_ = 38.52, *SD* = 12.12] and 225 Japanese participants [40.9% female, *M*_age_ = 42.32, *SD* = 8.92]).

#### 2.1.2. Materials and procedure

After consenting, participants completed questionnaires in the order of their recent stressful events, their friend’s instrumentality, support provision, and motivations for support provision. We then asked them to report their demographic information (gender, age, nationality, ethnicity, education attainment, annual income, the MacArthur scale of subjective social status).

Participants were initially asked to briefly describe the biggest stressor they had faced within the last three months and identify the most relevant type of stressor from nine options (family relationship, friend relationship, romantic relationship, academic, health, financial, job, future, or other). In addition, they were asked to rate how they felt about the stressful events by using five items (stressful, negative, responsible, resolvable, and controllable) with a seven-point Likert scale (0 = not at all, 6 = very much).

The participants were then asked to think of the friend who was closest to them, write his or her initials, and rate how useful or disruptive the friend was to achieve their goals in five domains (job and academic, hobby, social relationship, financial, and other important goals) using a seven-point scale (−3 = very disruptive, 0 = neither disruptive nor helpful, 3 = very helpful). The questions regarding the friend’s instrumentality were developed based on Ohtsubo and Yagi (2015). Because the internal consistency of the five items was acceptable (Cronbach’s α was 0.74 for Japanese and 0.76 for European Americans), the average was computed for each participant as the friend’s instrumentality score.

Next, the participants were asked to suppose that the friend was experiencing the stressful event they described previously and report on a seven-point scale (0 = never likely to do so, 6 = very likely to do so) to what extent they would help the friend in ways that they provide emotional support (three items, e.g., “I would encourage the friend by saying things like, ‘Do not worry, it’s going to be all right.’”; Cronbach’s α was 0.72 for Japanese and 0.63 for European Americans), instrumental support (three items, e.g., “I would suggest how to solve the problem to the friend.”; Cronbach’s α was 0.90 for Japanese and 0.84 for European Americans), companionship (three items, e.g., “I would increase the time I spend with the friend without talking about his/her problem.”; Cronbach’s α was 0.83 for Japanese and 0.84 for European Americans), and attentiveness (three items, e.g., “I would keep a little distance until the friend felt better, although I cared if he/she is okay.”; Cronbach’s α was 0.66 for Japanese and 0.64 for European Americans).

The participants were also presented with a list of items consisting of the six types of motivations for support provision and asked to report on a seven-point scale (0 = not at all, 6 = very much) how important each item would be to them when they thought about the ways they would help the friend. The six types of motivations were: (a) motivation for self-esteem, with three items (e.g., “I want the friend to be able to feel good about him/herself”) developed by Chen et al. (2012); (b) motivation for closeness, with three items (e.g., “I want the friend to feel close to me”) developed by Chen et al. (2012); (c) motivation for relational concern, with 11 items (e.g., “I do not want to hurt the friend more by asking about a problem he/she is having”), which we developed by adapting the related items used in Taylor et al. (2004); (d) motivation for the friend’s self-improvement, with six items (e.g., “I want to give the friend some time alone to calm down and reflect”); (e) low efficacy of helping, with three items (e.g., “I do not know how to react to the friend who is having trouble”); and (f) no interest in helping, with two items (e.g., “I want to avoid any trouble”). We conducted an exploratory factor analysis for all the items regarding motivations of support provision and obtained six factors after excluding eight items with lower factor loadings. As we expected, relational concern was divided into two factors: concern for an entire group (four items, Cronbach’s α was 0.77 for Japanese and 0.76 for European Americans) and concern for a friend (four items, Cronbach’s α was 0.85 for Japanese and 0.79 for European Americans). Additionally, the factors of self-esteem (three items, Cronbach’s α was 0.81 for Japanese and 0.84 for European Americans), closeness (three items, Cronbach’s α was 0.84 for Japanese and 0.85 for European Americans), self-improvement (three items, Cronbach’s α was 0.71 for Japanese and 0.62 for European Americans), and low efficacy (three items, Cronbach’s α was 0.85 for Japanese and 0.82 for European Americans) were included. The details of the factor analysis are presented in the Supplementary materials.

### 2.2. Results

#### 2.2.1. Characteristics of stressful events and relationship instrumentality with friends

Japanese rated their stressors as stressful, responsible, and controllable, as did European Americans (stressful: *M* (*SD*) s = 6.20 (0.87) vs. 6.10 (0.99), *t*(431) = 1.11, *p* = 0.27, responsible: *M* (*SD*)s = 3.98 (1.88) vs. 3.77 (2.10), *t*(431) = 1.09, *p* = 0.28, controllable: *M* (*SD*)s = 3.08 (1.51) vs. 2.83 (1.69), *t*(431) = 1.61, *p* = 0.11). However, Japanese (*M* = 5.84, *SD* = 1.33) rated their stressors as more negative than did European Americans (*M* = 5.28, *SD* = 1.82), *t*(431) = 3.62, *p* < 0.001, whereas European Americans (*M* = 3.74, *SD* = 2.08) felt more solvable than did Japanese (*M* = 3.07, *SD* = 1.62), *t*(431) = 3.77, *p* < 0.001. Participants in both cultures reported more stressful events in family relationships, health, financial circumstances, and work. In particular, the ratio of family relationships was higher in Japanese (31.1%) than in European Americans (17.8%), *X*^2^ (1, *N* = 433) = 9.61, *p* = 0.002. In contrast, European Americans were more likely than Japanese to report more stressful events in romantic relationships (6.7% vs. 0.4%, *X*^2^ (1, *N* = 433) = 5.73, *p* = 0.017) and academic matters (3.4% vs. 0.0%, *X*^2^ (1, *N* = 433) = 10.96, *p* < 0.001), although the ratio of the two stressors was relatively much lower in both cultures. Moreover, the mean score of the friend’s instrumentality was higher in European Americans (*M* = 1.27, *SD* = 1.33) than in Japanese (*M* = 0.87, *SD* = 1.12), *t*(431) = 5.20, *p* < 0.001.

#### 2.2.2. Support provision

We performed a 2 (culture) × 4 (support type: instrumental support, emotional support, companionship, and attentiveness) ANCOVA including the negative and solvable ratings for stressors and the friend’s instrumentality, which differed across cultures, as covariates.^1^ The main effects of culture and support type were significant, *F*(1, 428) = 24.92, *p* < 0.001, *η_p_*^2^ = 0.23, and *F*(3, 1,284) = 11.20, *p* < 0.001, *η_p_*^2^ = 0.03. Also, the culture and type of support interaction was significant, *F*(3, 1,284) = 56.54, *p* < 0.001, *η_p_*^2^ = 0.12. A post-hoc analysis showed that European Americans provided more emotional support, instrumental support, and companionship than did Japanese (emotional support: *F*(1, 428) = 86.76, *p* < 0.001, *η_p_*^2^ = 0.17, LS means (SE) = 4.20 (0.09) vs. 3.07 (0.08), instrumental support: *F*(1, 428) = 10.68, *p* = 0.001, *η_p_*^2^ = 0.02, LS means (SE) = 3.84 (0.09) vs. 3.41 (0.09), companionship: *F*(1, 428) = 37.92, *p* < 0.001, *η_p_*^2^ = 0.08, LS means (SE) = 3.99 (0.09) vs. 3.22 (0.09)), whereas Japanese provided more attentiveness than European Americans, *F*(1, 428) = 38.62, *p* < 0.001, *η_p_*^2^ = 0.08, LS means (SE) = 3.84 (0.08) vs. 3.15 (0.08). European Americans provided more emotional support than the three other types of support (*p*s < 0.001), whereas Japanese provided more attentiveness than the three other types of support (*p*s < 0.001).

#### 2.2.3. Motivation for support provision

We performed a 2 (culture) × 6 (motivation: self-esteem, closeness, concern for an entire group, concern for a friend, self-improvement, and low efficacy) ANCOVA including the negative and solvable ratings for stressors and the friend’s instrumentality as covariates. The main effects of culture and motivation were significant, *F*(1, 428) = 10.33, *p* = 0.001, *η_p_*^2^ = 0.02, and *F*(5, 2,140) = 5.09, *p* < 0.001, *η_p_*^2^ = 0.01. The culture and motivation interaction was also significant, *F*(5, 2,140) = 110.40, *p* < 0.001, *η_p_*^2^ = 0.21. A post-hoc analysis showed that European Americans reported greater motivation for self-esteem (*F*(1, 428) = 57.34, *p* < 0.001, *η_p_*^2^ = 0.12, LS means (SE) = 4.54 (0.09) vs. 3.59 (0.09)) and closeness (*F*(1, 428) = 127.01, *p* < 0.001, *η_p_*^2^ = 0.23, LS means (SE) = 4.69 (0.09) vs. 3.29 (0.08)) than Japanese. In contrast, Japanese reported greater motivation for concern for an entire group (*F*(1, 428) = 60.57, *p* < 0.001, *η_p_*^2^ = 0.12, LS means (SE) = 1.81 (0.07) vs. 0.97 (0.07)), concern for a friend (*F*(1, 428) = 45.91, *p* < 0.001, *η_p_*^2^ = 0.10, LS means (SE) = 4.08 (0.09) vs. 3.19 (0.09)), self-improvement (*F*(1, 428) = 48.31, *p* < 0.001, *η_p_*^2^ = 0.10, LS means (SE) = 2.92 (0.08) vs. 2.13 (0.08)), and low efficacy (*F*(1, 428) = 97.60, *p* < 0.001, *η_p_*^2^ = 0.19, LS means (SE) = 2.69 (0.09) vs. 1.43 (0.09)) than did European Americans. Concern for a friend was higher than self-esteem and closeness in Japanese (*p*s < 0.001), whereas the trends were reversed in European Americans (*p*s < 0.001). Overall, motivation for concern for an entire group was lower than the five other types of motivation in both cultures (*p*s < 0.001).

Table 1 presents the correlations between motivation and support provision in each culture. In both cultures, self-esteem and closeness were positively associated with the four types of support provision, whereas concern for a friend and self-improvement were positively associated with the two types of implicit support provision. Concern for an entire group and low efficacy were positively associated with providing attentiveness, whereas low efficacy was negatively associated with providing emotional support. Only in Japanese was concern for an entire group positively associated with providing companionship, and self-improvement was positively associated with the two types of explicit support provision. Additionally, low efficacy was positively associated with providing companionship but negatively associated with providing instrumental support. In contrast, concern for an entire group was negatively associated with providing emotional support only in European Americans.

**Table 1:** Correlations among support provision and motivation variables in Study 1.

Variable	1	2	3	4	5	6	7	8	9	10
1. Emotional support	––	0.48^***^	0.23^***^	0.19^**^	0.54^***^	0.54^***^	−0.05	−0.03	0.18^**^	−0.31^***^
2. Instrumental support	0.56^***^	––	−0.04	0.04	0.33^***^	0.31^***^	−0.13^+^	−0.13^+^	0.20^**^	−0.41^***^
3. Companionship	0.41^***^	0.36^***^	––	0.53^***^	0.26^***^	0.32^***^	0.26^***^	0.48^***^	0.19^**^	0.17^**^
4. Attentiveness	0.32^***^	0.24^***^	0.39^***^	––	0.32^***^	0.18^**^	0.14^*^	0.52^***^	0.31^***^	0.20^**^
5. Self-esteem	0.42^***^	0.35^***^	0.41^***^	0.23^***^	––	0.51^***^	0.11^+^	0.31^***^	0.42^***^	−0.12^+^
6. Closeness	0.53^***^	0.40^***^	0.47^***^	0.18^**^	0.63^***^	––	0.10	0.17^**^	0.18^***^	−0.02
7. Concern for group	−0.15^*^	0.01	0.02	0.25^***^	−0.001	−0.17^*^	––	0.34^***^	0.30^***^	0.52^***^
8. Concern for friend	0.13^+^	0.05	0.37^***^	0.41^***^	0.34^***^	0.13^+^	0.29^***^	––	0.23^***^	0.38^***^
9. Self-improvement	−0.06	0.03	0.16^*^	0.38^***^	0.19^**^	−0.02	0.47^***^	0.40^***^	––	0.07
10. Low efficacy	−0.20^**^	−0.12^+^	−0.10	0.20^**^	−0.01	−0.17^*^	0.57^***^	0.32^***^	0.37^***^	––

Correlations for Japanese (*df* = 223) are presented above the diagonal, and correlations for Americans (*df* = 206) are presented below the diagonal.

^***^p < 0.001, ^**^p < 0.01, ^*^p < 0.05, ^+^*p* < 0.10.

#### 2.2.4. Relationship between motivation and explicit support provision: self-esteem and closeness

We initially performed a series of multiple regression analyses to investigate whether specific types of motivations were associated with providing explicit support (i.e., emotional support and instrumental support) and whether the associations were moderated by culture. Following Chen et al. (2012), we analyzed the relationships between each of the two types of explicit support and each of the corresponding motivations (i.e., self-esteem and closeness) and investigated the unique effects of the two types of motivation by controlling for each other in the regression analyses.

##### 2.2.4.1. Self-esteem

For each explicit support, motivation for closeness was entered along with the control variables of the negative and solvable ratings for stressors, the friend’s instrumentality, gender, and age (Step 1). Culture (0 = Japanese, 1 = European Americans) and motivation for self-esteem were also entered (Step 2). The interaction between culture and motivation for self-esteem was then added (Step 3). For emotional support, the main effect of motivation for closeness was significant, *b* = 0.52, *SE* = 0.04, *p* < 0.001 in Step 1 (*R*^2^ = 0.474). Whereas the main effect of closeness was still significant, the main effects of motivation for self-esteem (*b* = 0.23, *SE* = 0.05, *p* < 0.001) and culture (*b* = 0.40, *SE* = 0.12, *p* < 0.001) were significant in Step 2 (Δ*R*^2^ = 0.042). Additionally, the culture and motivation for self-esteem interaction was significant, *b* = −0.17, *SE* = 0.08, *p* = 0.03 in Step 3 (Δ*R*^2^ = 0.006). In both cultures, motivation for self-esteem promoted more emotional support provision. However, the trend was rather stronger in Japanese (*b* = 0.32, *SE* = 0.06, *p* < 0.001) than in European Americans (*b* = 0.15, *SE* = 0.06, *p* = 0.01), contrary to Chen et al. (2012). For instrumental support, the main effect of motivation for closeness was significant, *b* = 0.32, *SE* = 0.04, *p* < 0.001 in Step 1 (*R*^2^ = 0.207). Whereas the main effect of closeness was still significant, the main effect of motivation for self-esteem (*b* = 0.20, *SE* = 0.06, *p* < 0.001) was significant in Step 2 (Δ*R*^2^ = 0.023). The culture and motivation for self-esteem interaction was not significant in Step 3, *b* = 0.0002, *SE* = 0.09, *p* = 0.999, suggesting that culture does not affect the increase of instrumental support by motivation for self-esteem.

##### 2.2.4.2. Closeness

For each explicit support, motivation for self-esteem was entered along with the control variables of the negative and solvable ratings for stressors, the friend’s instrumentality, gender, and age (Step 1). Culture and motivation for closeness were also entered (Step 2). The interaction between culture and motivation for closeness was then added (Step 3). For emotional support, the main effect of motivation for self-esteem was significant, *b* = 0.47, *SE* = 0.04, *p* < 0.001 in Step 1 (*R*^2^ = 0.421). Whereas the main effect of self-esteem was still significant, the main effects of motivation for closeness (*b* = 0.32, *SE* = 0.05, *p* < 0.001) and culture (*b* = 0.40, *SE* = 0.12, *p* < 0.001) were significant in Step 2 (Δ*R*^2^ = 0.095). The culture and motivation for closeness interaction was not significant in Step 3, *b* = −0.10, *SE* = 0.08, *p* = 0.22. For instrumental support, the main effect of motivation for self-esteem was significant, *b* = 0.33, *SE* = 0.05, *p* < 0.001 in Step 1 (*R*^2^ = 0.204). Whereas the main effect of self-esteem was still significant, the main effect of motivation for closeness (*b* = 0.21, *SE* = 0.06, *p* < 0.001) was significant in Step 2 (Δ*R*^2^ = 0.026). The culture and motivation for closeness was not significant in Step 3, *b* = 0.06, *SE* = 0.09, *p* = 0.54. In sum, motivation for closeness increased both types of explicit support, regardless of culture.

##### 2.2.4.3. Mediation analysis

**Figure 2:**
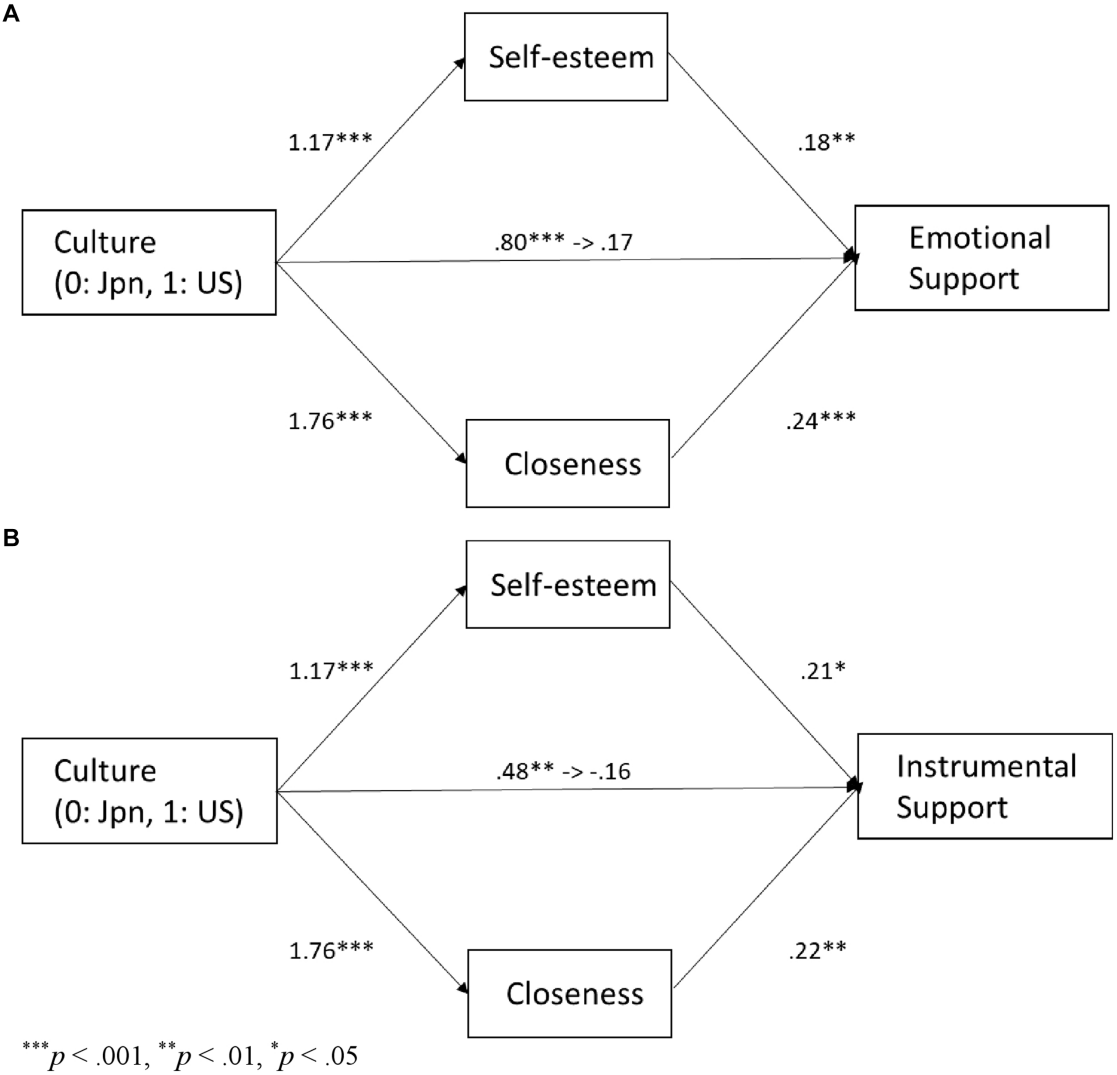
The relationship between culture and explict support provision, mediated by motivation for self-esteem, and closeness in Study 1: emotional support **(A)** and instrumental support **(B)**.

As shown in Table 1, motivation for self-esteem and motivation for closeness were highly positively correlated with explicit support provision in both cultures. Additionally, explicit support provision and both types of motivation were significantly higher in European Americans than in Japanese. These patterns suggest that both types of motivation function as mediators. To examine whether cultural influences on emotional support provision were mediated by motivation for self-esteem and motivation for closeness, we conducted a mediation analysis using a bootstrapping test with 2,000 replications. Culture (0 = Japanese, 1 = European Americans) was positively associated with motivation for self-esteem (*b* = 1.17, *SE* = 0.15, *p* < 0.001), motivation for closeness (*b* = 1.76, *SE* = 0.15, *p* < 0.001), and emotional support provision (*b* = 0.80, *SE* = 0.11, *p* < 0.001). The path from culture to emotional support provision became non-significant when motivation for self-esteem and motivation for closeness were entered as joint predictors of emotional support provision, *b* = 0.17, *SE* = 0.11, *p* = 0.11. Motivation for self-esteem (*b* = 0.18, *SE* = 0.05, *p* = 0.001) and motivation for closeness (*b* = 0.24, *SE* = 0.06, *p* < 0.001) predicted emotional support provision (Figure 2A). The indirect effect through motivation for self-esteem (95% bias-corrected CI = [0.09, 0.37]) and through motivation for closeness (95% bias-corrected CI = [0.22, 0.69]) were significant. We also conducted a comparable analysis regarding instrumental support provision. Culture was positively associated with instrumental support provision (*b* = 0.48, *SE* = 0.14, *p* = 0.001). The path became non-significant when motivation for self-esteem and motivation for closeness were entered as joint predictors of instrumental support provision, *b* = −0.16, *SE* = 0.17, *p* = 0.33. Motivation for self-esteem (*b* = 0.21, *SE* = 0.08, *p* = 0.01) and motivation for closeness (*b* = 0.22, *SE* = 0.07, *p* = 0.002) predicted instrumental support provision (Figure 2B). The indirect effect through motivation for self-esteem (95% bias-corrected CI = [0.06, 0.47]) and through motivation for closeness (95% bias-corrected CI = [0.14, 0.67]) were significant.

#### 2.2.5. Relationship between motivation and implicit support provision: concern for an entire group and concern for a friend

We then analyzed the relationships between each of the two types of implicit support (companionship and attentiveness) and each of the two types of relational concern (i.e., concern for an entire group and concern for a friend) in the same way as we did in terms of the relationship between motivation and explicit support provision.

##### 2.2.5.1. Concern for an entire group

**Figure 3:**
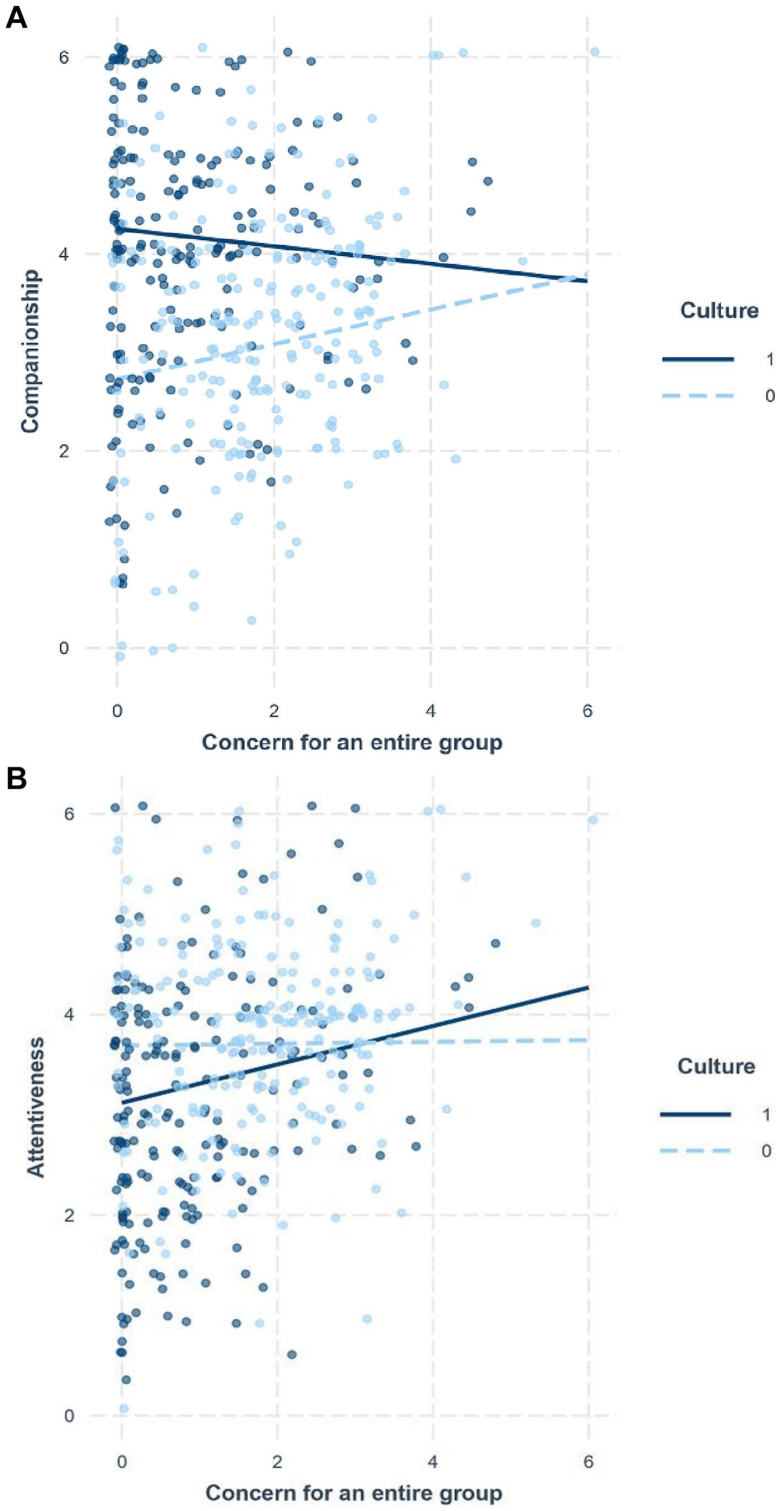
The moderating role of culture (0: Jpn, 1: US) in the relationship between concern for an entire group and implicit support provision in Study 1: companionship **(A)** and attentiveness **(B)**.

For each implicit support, concern for a friend was entered along with the control variables of the negative and solvable ratings for stressors, the friend’s instrumentality, gender, and age (Step 1). Culture and concern for an entire group were additionally entered (Step 2). The interaction between culture and concern for an entire group was then added (Step 3). For companionship, the main effect of concern for a friend was significant, *b* = 0.28, *SE* = 0.04, *p* < 0.001 in Step 1 (*R*^2^ = 0.152). Whereas the main effect of concern for a friend was still significant, the main effect of culture (*b* = 1.16, *SE* = 0.13, *p* < 0.001) was significant in Step 2 (Δ*R*^2^ = 0.095). Although the main effect of concern for an entire group was not significant in Step 2 (*b* = 0.06, *SE* = 0.05, *p* = 0.29), culture moderated the effect of concern for an entire group in Step 3 (Δ*R*^2^ = 0.012), *b* = −0.27, *SE* = 0.10, *p* = 0.009. Whereas greater concern for an entire group led Japanese to provide more companionship (*b* = 0.18, *SE* = 0.07, *p* = 0.01), the trend disappeared in European Americans (*b* = −0.09, *SE* = 0.08, *p* = 0.25) (Figure 3A). For attentiveness, the main effect of concern for a friend was significant, *b* = 0.42, *SE* = 0.04, *p* < 0.001 in Step 1 (*R*^2^ = 0.261). Whereas the main effect of concern for a friend was still significant, the main effect of concern for an entire group was not significant, *b* = 0.09, *SE* = 0.05, *p* = 0.053 in Step 2 (Δ*R*^2^ = 0.027). In contrast, the main effect of culture (*b* = −0.32, *SE* = 0.11, *p* = 0.004) was significant in Step 2. Additionally, culture moderated the effect of concern for an entire group in Step 3 (Δ*R*^2^ = 0.007), *b* = 0.18, *SE* = 0.09, *p* = 0.036. Contrary to our expectation, greater concern for an entire group likely led European Americans to provide more attentiveness (*b* = 0.19, *SE* = 0.07, *p* = 0.004), whereas the tendency disappeared in Japanese (*b* = 0.01, *SE* = 0.06, *p* = 0.88) (Figure 3B).

##### 2.2.5.2. Concern for a friend

For each implicit support, concern for an entire group was entered along with the control variables of the negative and solvable ratings for stressors, the friend’s instrumentality, gender, and age (Step 1). Culture and concern for a friend were additionally entered (Step 2). The interaction between culture and concern for a friend was then added (Step 3). For companionship, the main effect of concern for an entire group was not significant, *b* = 0.08, *SE* = 0.06, *p* = 0.14 in Step 1 (*R*^2^ = 0.075). The main effects of culture (*b* = 1.16, *SE* = 0.13, *p* < 0.001) and concern for a friend (*b* = 0.39, *SE* = 0.04, *p* < 0.001) were significant in Step 2 (Δ*R*^2^ = 0.215). The culture and concern for a friend interaction was not significant, *b* = −0.16, *SE* = 0.09, *p* = 0.058 in Step 3 (Δ*R*^2^ = 0.006). For attentiveness, the main effect of concern for an entire group was significant, *b* = 0.31, *SE* = 0.05, *p* < 0.001 in Step 1 (*R*^2^ = 0.101). The main effects of culture (*b* = −0.32, *SE* = 0.11, *p* = 0.004) and concern for a friend (*b* = 0.36, *SE* = 0.04, *p* < 0.001) were significant, whereas the main effect of concern for an entire group was found not to be significant, *b* = 0.09, *SE* = 0.05, *p* = 0.053 in Step 2 (Δ*R*^2^ = 0.186). The culture and concern for a friend interaction was not significant, *b* = −0.04, *SE* = 0.07, *p* = 0.62 in Step 3 (Δ*R*^2^ = 0.0004). In sum, greater concern for a friend led people to provide more both types of implicit support regardless of culture.

##### 2.2.5.3. Mediation analysis

**Figure 4:**
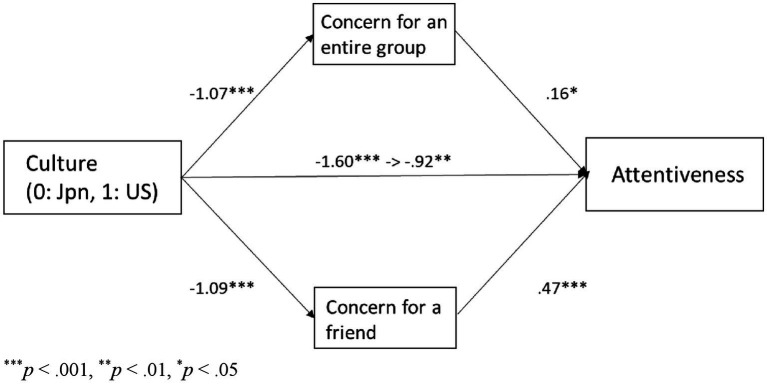
The relationship between culture and attentiveness provision mediated by concern for an entire group and concern for a friend in Study 1.

As shown in Table 1, concern for an entire group motivation and concern for a friend motivation were highly positively correlated with attentiveness provision in both cultures. Additionally, attentiveness provision and both types of motivation were significantly higher in Japanese than in European Americans. To examine whether cultural influences on attentiveness provision were mediated by concern for an entire group motivation and concern for a friend motivation, we conducted a mediation analysis by using a bootstrapping test with 2,000 replications. Culture (0 = Japanese, 1 = European Americans) was negatively associated with attentiveness provision (*b* = −1.60, *SE* = 0.24, *p* < 0.001), concern for an entire group motivation (*b* = −1.07, *SE* = 0.12, *p* < 0.001) and concern for a friend motivation (*b* = −1.09, *SE* = 0.14, *p* < 0.001). The path was still significant when concern for an entire group motivation and concern for a friend motivation were entered as joint predictors of attentiveness provision, *b* = −0.92, *SE* = 0.31, *p* = 0.003. Concern for an entire group motivation (*b* = 0.16, *SE* = 0.08, *p* = 0.03) and concern for a friend motivation (*b* = 0.47, *SE* = 0.11, *p* < 0.001) predicted attentiveness provision (Figure 4). The indirect effect through concern for an entire group motivation (95% bias-corrected CI = [−0.33, −0.007]) and that through concern for a friend motivation (95% bias-corrected CI = [−0.79, −0.23]) were significant.

## 3. Discussion

In summary, European Americans reported that they would provide more emotional support and instrumental support and have higher motivation for self-esteem and closeness compared to Japanese, consistent with Hypothesis 1. Study 1 also found that motivations for self-esteem and closeness positively influenced emotional and instrumental support provision in both cultures. The cultural differences in emotional support and instrumental support provision were thus mediated by self-esteem motivation and closeness motivation, which varied across cultures. This supports Hypothesis 2b but not Hypothesis 2a. Our moderation analysis showed that the relationship between emotional support and self-esteem motivation was stronger in Japanese than European Americans, which contradicts the findings of Chen et al. (2012).

In addition, consistent with Hypothesis 3, Japanese reported that they would provide more attentiveness support and have higher motivations for concern for an entire group and concern for a friend compared to European Americans. However, the results for companionship support were unexpected, as Japanese reported providing lower levels of this type of support. The relationships between concern for an entire group motivation and the provision of implicit social support differed between the two cultures. Hypothesis 4a was partially supported, as the relationship between companionship support and concern for an entire group motivation was stronger in Japanese than European Americans. However, the relationship between attentiveness support and concern for an entire group motivation was stronger in European Americans than in Japanese. Hypothesis 4b was also partially supported, as the cultural difference in attentiveness support was mediated by concern for an entire group motivation and concern for a friend motivation, which varied across cultures.

## 4. Study 2

To address a potential limitation of Study 1, in which participants may have projected the type of support they preferred to receive onto the support they would provide, Study 2 was conducted. In this study, a different group of Japanese and European American participants were asked to recall a recent stressful event experienced by a close other and report on the actual support provided and the underlying motivation. This procedure aimed to eliminate the possibility that the results in Study 1 were influenced by the participants’ desired support type.

### 4.1. Method

#### 4.1.1. Participants

Based on the results of the power analysis reported in Study 1, we recruited 300 European Americans and 342 Japanese participants through online crowdsourcing marketplaces (Prolific for the European American participants and Lancers for the Japanese participants). Sixty-three participants were excluded from the following analysis due to the following reasons: Six Japanese and two European American participants failed to pass attention check questions, 38 Japanese participants did not complete more than half of the whole questionnaire, one Japanese participant did not allow us to analyze their data, and five participants recruited in Japan chose another option than Asian regarding their ethnicity and 11 Americans chose another option than European American regarding their ethnicity. Thus, the final sample size was 579 (287 European Americans [46.0% female, *M*_age_ = 38.40, *SD* = 14.17] and 292 Japanese participants [44.2% female, *M*_age_ = 43.16, *SD* = 9.83]).

#### 4.1.2. Materials and procedure

After consenting, the participants completed questionnaires in the order of their close friend’s recent stressful events, the friend’s instrumentality, support provision, and motivations for support provision. We then asked them to report their demographic information (gender, age, nationality, ethnicity, education attainment, annual income, the MacArthur scale of subjective social status).

The participants were initially asked to briefly describe the biggest stressor their close friend had faced within the last three months and rate how they felt it by using two items (stressful and negative) on a seven-point Likert scale (0 = not at all, 6 = very much). They were then asked to write the initials of the friend and rate how useful or disruptive the friend was to achieve their goals using a seven-point scale (−3 = very disruptive, 0 = neither disruptive nor helpful, 3 = very helpful), as in Study 1. The internal consistency of the five items was acceptable (Cronbach’s α was 0.80 for Japanese and 0.81 for European Americans); the average was computed for each participant as the friend’s instrumentality score.

Next, the participants were asked to report on a seven-point scale (0 = never likely to do so, 6 = very likely to do so) to what extent they helped the friend in ways that they provided support. The 12 items used were identical to those used in Study 1. We conducted a confirmatory factor analysis of the 12-item scale for each culture. The items showed acceptable fit in both cultures (America: *χ*^2^ (48) = 107.66, *p* < 0.001, CFI = 0.953, RMSEA = 0.066; Japan: *χ*^2^(48) = 234.50, *p* < 0.001, CFI = 0.880, RMSEA = 0.115). All items loaded significantly on their target factors in both cultures (emotional support: Cronbach’s α was 0.72 for Japanese and 0.67 for European Americans, instrumental support: Cronbach’s α was 0.90 for Japanese and 0.88 for European Americans, companionship: Cronbach’s α was 0.77 for Japanese and 0.84 for European Americans, and attentiveness: Cronbach’s α was 0.75 for Japanese and 0.55 for European Americans). The details of the factor analysis are presented in the Supplementary materials.

The participants were also presented with a list of 20 items consisting of the 6 types of motivations of support provision and asked to report on a 7-point scale (0 = not at all, 6 = very much) how important each item would be to them when they helped the friend. The 20 items used were identical to those analyzed in Study 1. We conducted a confirmatory factor analysis of the 20-item scale for each culture. Because one item loaded weakly on a target factor (self-improvement), it was dropped from further analyses. The remaining 19 items showed acceptable fit in both cultures (America: *χ*^2^(137) = 260.18, *p* < 0.001, CFI = 0.946, RMSEA = 0.056, Japan: *χ*^2^(137) = 342.09, *p* < 0.001, CFI = 0.908, RMSEA = 0.072). All items loaded significantly on their target factors in both cultures (self-esteem: Cronbach’s α was 0.84 for Japanese and 0.84 for European Americans, closeness: Cronbach’s α was 0.84 for Japanese and 0.86 for European Americans, concern for an entire group: Cronbach’s α was 0.72 for Japanese and 0.81 for European Americans, concern for a friend: Cronbach’s α was 0.81 for Japanese and 0.76 for European Americans, self-improvement: Cronbach’s α was 0.70 for Japanese and 0.60 for European Americans, and low efficacy: Cronbach’s α was 0.76 for Japanese and 0.70 for European Americans). The details of the factor analysis are presented in the Supplementary materials.

### 4.2. Results

#### 4.2.1. Characteristics of stressful events and relationship instrumentality with friends

European Americans (*M* = 6.59, *SD* = 0.74) rated their friends’ stressors as more stressful than did Japanese (*M* = 6.28, *SD* = 0.93), *t*(577) = 4.42 *p* < 0.001, whereas there was no cultural difference in the rating of negativity (European Americans: *M* = 6.11, *SD* = 1.44, Japanese: *M* = 6.16, *SD* = 1.12), *t*(577) = 0.46, *p* = 0.64. Moreover, the mean score of the friend’s instrumentality was higher in European Americans (*M* = 0.87, *SD* = 1.04) than in Japanese (*M* = 0.71, *SD* = 0.91), *t*(577) = 2.03, *p* = 0.04.

#### 4.2.2. Support provision

We performed a 2 (culture) × 4 (support type: instrumental support, emotional support, companionship, and attentiveness) ANCOVA including the stressful rating for stressors and the friend’s instrumentality, which differed across cultures, as covariates.^2^ The main effect of support type was significant, *F*(3, 1725) = 3.94, *p* = 0.008, *η_p_*^2^ = 0.01. Also, the culture and type of support interaction was significant, *F*(3, 1725) = 45.82, *p* < 0.001, *η_p_*^2^ = 0.07. As in Study 1, a post-hoc analysis showed that European Americans provided more emotional support, instrumental support, and companionship than did Japanese (emotional support: *F*(1, 575) = 18.34, *p* < 0.001, *η_p_*^2^ = 0.03, LS means (SE) = 3.49 (0.08) vs. 2.97 (0.08), instrumental support: *F*(1, 575) = 12.56, *p* < 0.001, *η_p_*^2^ = 0.02, LS means (SE) = 3.37 (0.10) vs. 2.88 (0.10), companionship: *F*(1, 575) = 9.51, *p* = 0.002, *η_p_*^2^ = 0.02, LS means (SE) = 3.34 (0.09) vs. 2.96 (0.09)), whereas Japanese provided more attentiveness than did European Americans, *F*(1, 575) = 82.58, *p* < 0.001, *η_p_*^2^ = 0.13, LS means (SE) = 3.67 (0.07) vs. 2.73 (0.07). European Americans provided more emotional support than the three other types of support (*p*s < 0.001), whereas Japanese provided more attentiveness than the three other types of support (*p*s < 0.001).

#### 4.2.3. Motivation for support provision

We performed a 2 (culture) × 6 (motivation: self-esteem, closeness, concern for an entire group, concern for a friend, self-improvement, and low efficacy) ANCOVA including the stressful rating for stressors and the friend’s instrumentality as covariates. The main effects of culture and motivation were significant, *F*(1, 575) = 35.55, *p* < 0.001, *η_p_*^2^ = 0.06, and *F*(5, 2,875) = 8.08, *p* < 0.001, *η_p_*^2^ = 0.01. The interaction between culture and motivation was also significant, *F*(5, 2,875) = 99.08, *p* < 0.001, *η_p_*^2^ = 0.15. As in Study 1, a post-hoc analysis showed that European Americans reported greater motivation for self-esteem (*F*(1, 575) = 32.54, *p* < 0.001, *η_p_*^2^ = 0.05, LS means (SE) = 4.27 (0.08) vs. 3.63 (0.08)) and closeness (*F*(1, 575) = 54.92, *p* < 0.001, *η_p_*^2^ = 0.09, LS means (SE) = 4.16 (0.08) vs. 3.33 (0.08)) than did Japanese. In contrast, Japanese reported greater motivation for concern for an entire group (*F*(1, 575) = 68.13, *p* < 0.001, *η_p_*^2^ = 0.11, LS means (SE) = 1.66 (0.06) vs. 0.89 (0.06)), concern for a friend (*F*(1, 575) = 101.21, *p* < 0.001, *η_p_*^2^ = 0.15, LS means (SE) = 3.76 (0.08) vs. 2.60 (0.08)), self-improvement (*F*(1, 575) = 20.49, *p* < 0.001, *η_p_*^2^ = 0.03, LS means (SE) = 2.46 (0.08) vs. 1.92 (0.08)), and low efficacy (*F*(1, 575) = 168.28, *p* < 0.001, *η_p_*^2^ = 0.23, LS means (SE) = 3.12 (0.08) vs. 1.62 (0.08)) than did European Americans. Concern for a friend was higher than self-esteem and closeness in Japanese (*p*s < 0.001), whereas the trends were reversed in European Americans (*p*s < 0.001). Overall, the motivation for concern for an entire group was lower than the five other types of motivation in both cultures (*p*s < 0.001).

Table 2 presents the correlations between motivation and support provision in each culture. Consistent with Study 1, self-esteem and closeness were positively associated with the four types of support provision, whereas concern for a friend and self-improvement were positively associated with the two types of implicit support provision in both cultures. Additionally, in both cultures, providing instrumental support was positively linked to self-improvement but negatively linked to low efficacy. Consistent with Study 1, concern for an entire group was positively associated with providing companionship only in Japanese. Additionally, it was specific to Japanese that emotional support was positively linked to concern for a friend and self-improvement but negatively linked to low efficacy. In contrast, concern for an entire group and low efficacy were positively associated with providing attentiveness only in European Americans.

**Table 2:** Correlations among support provision and motivation variables in Study 2.

Variable	1	2	3	4	5	6	7	8	9	10
1. Emotional support	––	0.47^***^	0.33^***^	0.43^***^	0.52^***^	0.58^***^	−0.09	0.16^**^	0.18^**^	−0.17^**^
2. Instrumental support	0.50^***^	––	0.04	0.14^*^	0.34^***^	0.35^***^	−0.05	−0.11^+^	0.18^**^	−0.47^***^
3. Companionship	0.43^***^	0.25^***^	––	0.39^***^	0.34^***^	0.39^***^	0.23^***^	0.38^***^	0.27^***^	0.06
4. Attentiveness	0.27^***^	0.08	0.22^***^	––	0.41^***^	0.36^***^	0.05	0.42^***^	0.22^***^	0.02
5. Self-esteem	0.47^***^	0.38^***^	0.34^***^	0.18^**^	––	0.57^***^	0.02	0.38^***^	0.44^***^	−0.08
6. Closeness	0.53^***^	0.25^***^	0.39^***^	0.19^**^	0.64^***^	––	0.01	0.24^***^	0.27^***^	−0.10
7. Concern for group	0.03	0.07	0.05	0.32^***^	−0.001	−0.07	––	0.37^***^	0.43^***^	0.36^***^
8. Concern for friend	0.11^+^	−0.003	0.24^***^	0.40^***^	0.26^***^	0.22^***^	0.44^***^	––	0.34^***^	0.31^***^
9. Self-improvement	−0.01	0.16^**^	0.13^*^	0.30^***^	0.13^*^	0.01	0.46^***^	0.40^***^	––	0.16^**^
10. Low efficacy	−0.09	−0.22^***^	0.01	0.30^***^	−0.03	−0.02	0.57^***^	0.52^***^	0.29^***^	––

Correlations for Japanese (*df* = 290) are presented above the diagonal, and correlations for Americans (*df* = 285) are presented below the diagonal.

^***^p < 0.001, ^**^p < 0.01, ^*^p < 0.05, ^+^*p* < 0.10.

#### 4.2.4. Relationship between motivation and explicit support provision: self-esteem and closeness

As in Study 1, we analyzed the relationships between each of the two types of explicit support (i.e., emotional support and instrumental support) and each of the corresponding motivations (i.e., self-esteem and closeness) and investigated the unique effects of the two types of motivation and the moderating role of culture by controlling for each other in the regression analyses.

##### 4.2.4.1. Self-esteem

For each explicit support, motivation for closeness was entered along with the control variables of the stressful rating for stressors, the friend’s instrumentality, gender, and age (Step 1). Culture (0 = Japanese, 1 = European Americans) and motivation for self-esteem were also entered (Step 2). The interaction between culture and motivation for self-esteem was then added (Step 3). For emotional support, the main effect of motivation for closeness was significant, *b* = 0.52, *SE* = 0.04, *p* < 0.001 in Step 1 (*R*^2^ = 0.360). Whereas the main effect of closeness was still significant, the main effect of motivation for self-esteem (*b* = 0.25, *SE* = 0.05, *p* < 0.001) was significant in Step 2 (Δ*R*^2^ = 0.033). The culture and motivation for self-esteem interaction was not significant in Step 3, *b* = −0.05, *SE* = 0.07, *p* = 0.53 (Δ*R*^2^ = 0.0004). In both cultures, motivation for self-esteem promoted more emotional support provision. For instrumental support, the main effect of motivation for closeness was significant, *b* = 0.33, *SE* = 0.05, *p* < 0.001 in Step 1 (*R*^2^ = 0.127). Whereas the main effect of closeness was still significant, the main effect of motivation for self-esteem (*b* = 0.34, *SE* = 0.06, *p* < 0.001) was significant in Step 2 (Δ*R*^2^ = 0.052). The culture and motivation for self-esteem was not significant in Step 3, *b* = −0.14, *SE* = 0.09, *p* = 0.13, suggesting that in both cultures, motivation for self-esteem also increased instrumental support provision.

##### 4.2.4.2. Closeness

For each social support, motivation for self-esteem was entered along with the control variables of the stressful rating for stressors, the friend’s instrumentality, gender, and age (Step 1). Culture and motivation for closeness were also entered (Step 2). The interaction between culture and motivation for closeness was then added (Step 3). For emotional support, the main effect of motivation for self-esteem was significant, *b* = 0.48, *SE* = 0.04, *p* < 0.001 in Step 1 (*R*^2^ = 0.318). Whereas the main effect of self-esteem was still significant, the main effect of motivation for closeness (*b* = 0.38, *SE* = 0.05, *p* < 0.001) was significant in Step 2 (Δ*R*^2^ = 0.076). The culture and motivation for closeness interaction was not significant in Step 3, *b* = −0.08, *SE* = 0.07, *p* = 0.29. For instrumental support, the main effect of motivation for self-esteem was significant, *b* = 0.42, *SE* = 0.05, *p* < 0.001 in Step 1 (*R*^2^ = 0.171). Whereas the main effect of self-esteem was still significant, the main effect of motivation for closeness (*b* = 0.12, *SE* = 0.06, *p* = 0.04) was significant in Step 2 (Δ*R*^2^ = 0.009). The culture and motivation for closeness relationship was not significant in Step 3, *b* = −0.14, *SE* = 0.09, *p* = 0.13. In sum, motivation for closeness increased both types of explicit support, regardless of culture.

##### 4.2.4.3. Mediation analysis

**Figure 5:**
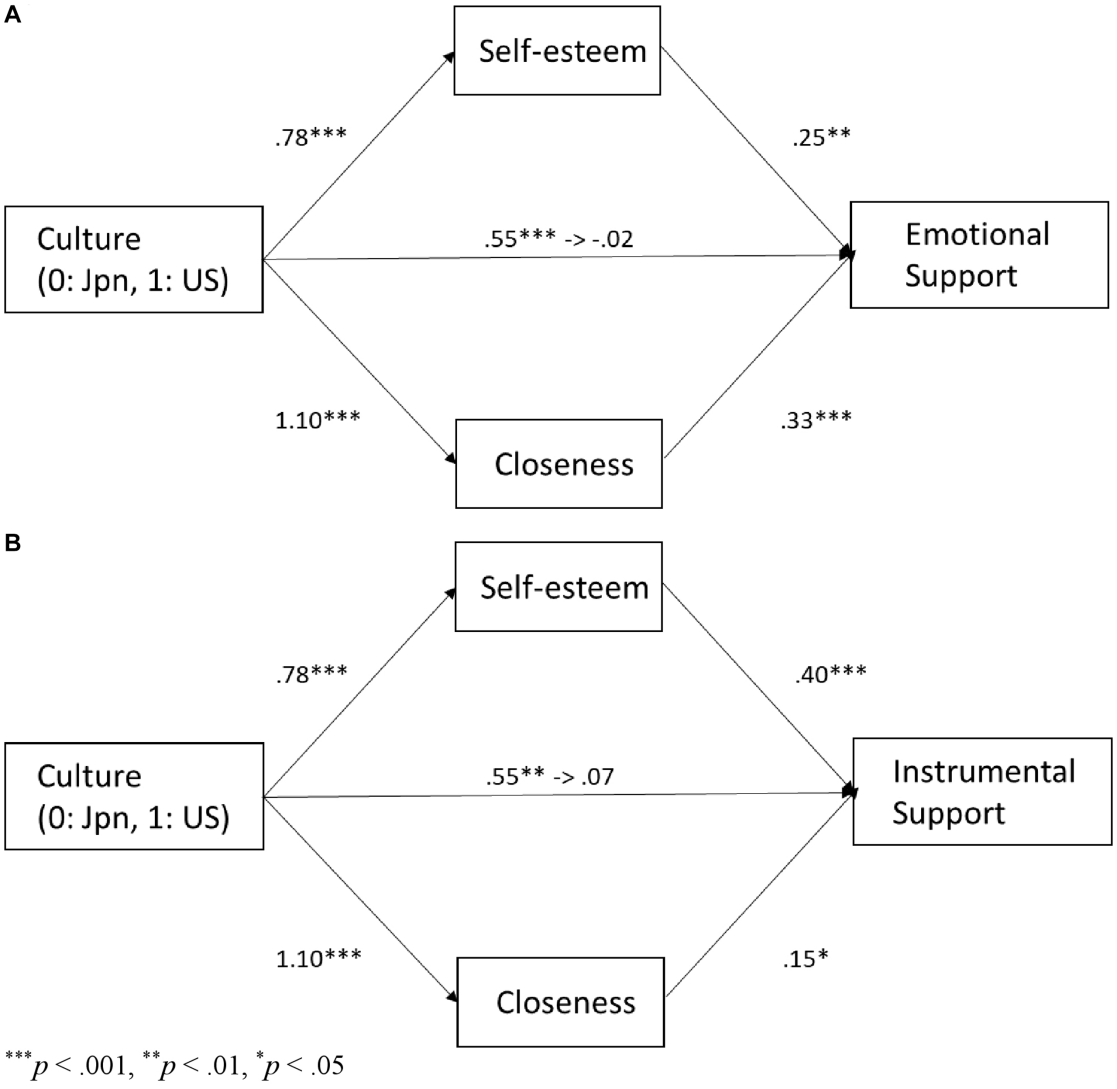
The relationship between culture and explicit support provision, mediated by motivation for self-esteem and closeness in Study 2: emotional support **(A)** and instrumental support **(B)**.

As shown in Table 2, motivation for self-esteem and motivation for closeness were highly positively correlated with explicit support provision in both cultures. Additionally, explicit support provision and both types of motivation were significantly higher in European Americans than in Japanese. These patterns were consistent with Study 1. As in Study 1, we conducted a mediation analysis by using a bootstrapping test with 2,000 replications to examine whether motivation for self-esteem and motivation for closeness function as mediators. Culture (0 = Japanese, 1 = European Americans) was positively associated with motivation for self-esteem (*b* = 0.78, *SE* = 0.12, *p* < 0.001), motivation for closeness (*b* = 1.10, *SE* = 0.14, *p* < 0.001), and emotional support provision (*b* = 0.55, *SE* = 0.10, *p* < 0.001). The path from culture to emotional support provision became non-significant when motivation for self-esteem and motivation for closeness were entered as joint predictors of emotional support provision, *b* = −0.02, *SE* = 0.10, *p* = 0.87. Motivation for self-esteem (*b* = 0.25, *SE* = 0.06, *p* = 0.001) and motivation for closeness (*b* = 0.33, *SE* = 0.05, *p* < 0.001) predicted emotional support provision (Figure 5A). The indirect effect through motivation for self-esteem (95% bias-corrected CI = [0.10, 0.31]) and through motivation for closeness (95% bias-corrected CI = [0.23, 0.54]) were significant. We also conducted a comparable analysis regarding instrumental support provision. Culture was positively associated with instrumental support provision (*b* = 0.55, *SE* = 0.15, *p* = 0.001). The path became non-significant when motivation for self-esteem and motivation for closeness were entered as joint predictors of instrumental support provision, *b* = 0.07, *SE* = 0.15, *p* = 0.64. Motivation for self-esteem (*b* = 0.40, *SE* = 0.08, *p* < 0.001) and motivation for closeness (*b* = 0.15, *SE* = 0.07, *p* = 0.02) predicted instrumental support provision (Figure 5B). The indirect effect through motivation for self-esteem (95% bias-corrected CI = [0.18, 0.47]) and through motivation for closeness (95% bias-corrected CI = [0.02, 0.32]) were significant.

#### 4.2.5. Relationship between motivation and implicit support provision: concern for an entire group and concern for a friend

As in Study 1, we also analyzed relationships between each of the two types of implicit support (companionship and attentiveness) and each of the two types of relational concern (i.e., concern for an entire group and concern for a friend).

##### 4.2.5.1. Concern for an entire group

**Figure 6:**
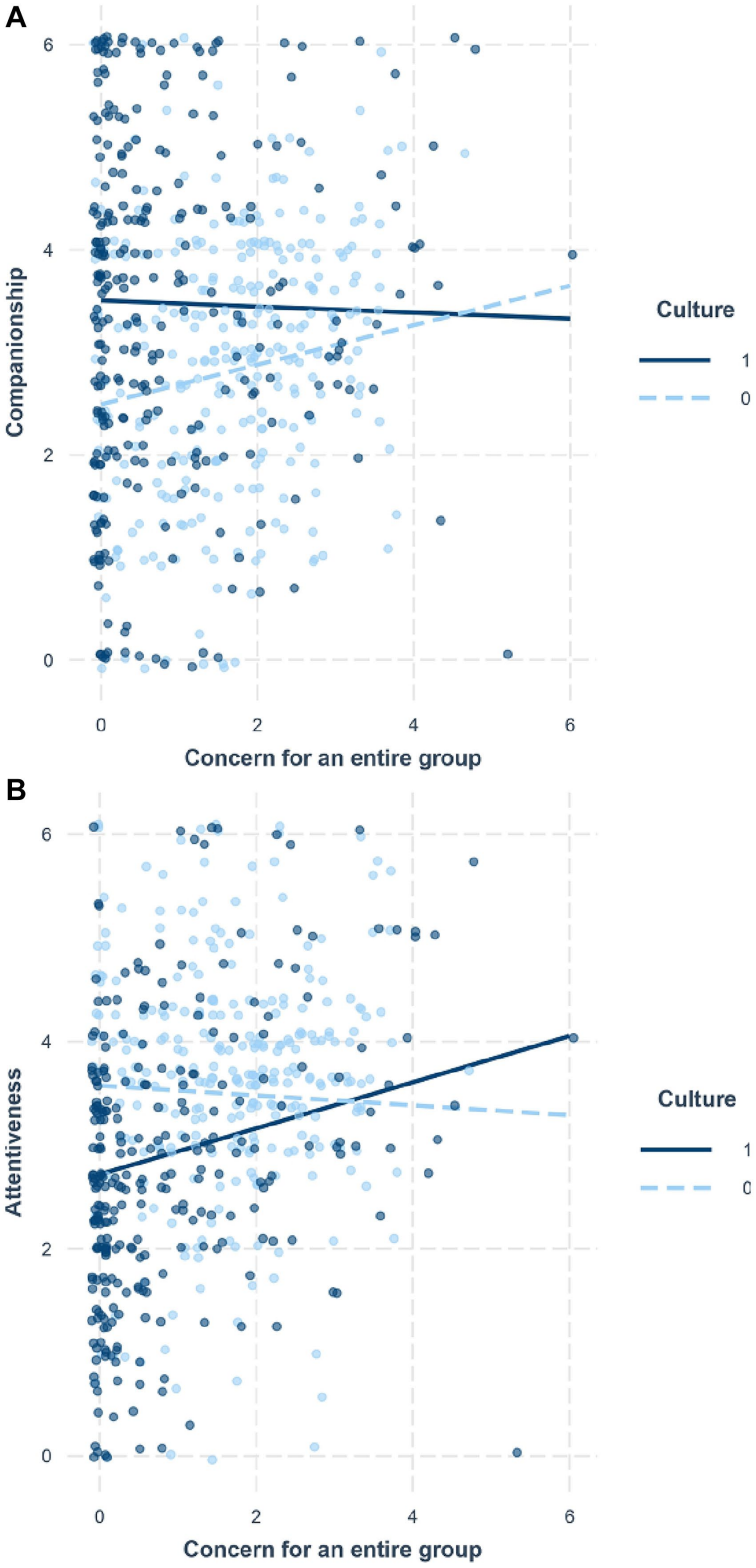
The moderating role of culture (0: Jpn, 1: US) in the relationship between concern for an entire group and implicit support provision in Study 2: companionship **(A)** and attentiveness **(B)**.

For each implicit support, concern for a friend was entered along with the control variables of the stressful rating for stressors, the friend’s instrumentality, gender, and age (Step 1). Culture and concern for an entire group were additionally entered (Step 2). The interaction between culture and concern for an entire group was then added (Step 3). For companionship, the main effect of concern for a friend was significant, *b* = 0.21, *SE* = 0.04, *p* < 0.001 in Step 1 (*R*^2^ = 0.118). Whereas the main effect of concern for a friend was still significant, the main effect of culture (*b* = 0.71, *SE* = 0.13, *p* < 0.001) was significant in Step 2 (Δ*R*^2^ = 0.043). Although the main effect of concern for an entire group was not significant in Step 2 (*b* = 0.07, *SE* = 0.06, *p* = 0.23), culture moderated the effect of concern for an entire group in Step 3 (Δ*R*^2^ = 0.006), *b* = −0.22, *SE* = 0.11, *p* = 0.04. Consistent with Study 1, whereas greater concern for an entire group led Japanese to provide more companionship (*b* = 0.19, *SE* = 0.08, *p* = 0.02), the trend disappeared in European Americans (*b* = −0.03, *SE* = 0.08, *p* = 0.70) (Figure 6A). For attentiveness, the main effect of concern for a friend was significant, *b* = 0.43, *SE* = 0.03, *p* < 0.001 in Step 1 (*R*^2^ = 0.262). Whereas the main effect of concern for a friend was still significant, the main effects of culture (*b* = −0.50, *SE* = 0.11, *p* < 0.001) and concern for an entire group were significant (*b* = 0.10, *SE* = 0.05, *p* = 0.04) in Step 2 (Δ*R*^2^ = 0.037). Additionally, culture moderated the effect of concern for an entire group in Step 3 (Δ*R*^2^ = 0.012), *b* = 0.27, *SE* = 0.08, *p* = 0.001. Consistent with Study 1, greater concern for an entire group likely led European Americans to provide more attentiveness (*b* = 0.22, *SE* = 0.06, *p* < 0.001), whereas the tendency disappeared in Japanese (*b* = −0.05, *SE* = 0.07, *p* = 0.47) (Figure 6B).

##### 4.2.5.2. Concern for a friend

For each implicit support, concern for an entire group was entered along with the control variables of the stressful rating for stressors, the friend’s instrumentality, gender, and age (Step 1). Culture and concern for a friend were additionally entered (Step 2). The interaction between culture and concern for a friend was then added (Step 3). For companionship, the main effect of concern for an entire group was significant, *b* = 0.15, *SE* = 0.05, *p* = 0.004 in Step 1 (*R*^2^ = 0.089). The main effects of culture (*b* = 0.71, *SE* = 0.13, *p* < 0.001) and concern for a friend (*b* = 0.27, *SE* = 0.05, *p* < 0.001) were significant, whereas the main effect of concern for an entire group was found not to be significant, *b* = 0.07, *SE* = 0.06, *p* = 0.23 in Step 2 (Δ*R*^2^ = 0.072). The culture and concern for a friend interaction was not significant, *b* = −0.09, *SE* = 0.09, *p* = 0.30 in Step 3 (Δ*R*^2^ = 0.002). For attentiveness, the main effect of concern for an entire group was significant, *b* = 0.38, *SE* = 0.04, *p* < 0.001 in Step 1 (*R*^2^ = 0.140). Whereas the main effect of concern for a friend was still significant, the main effects of culture (*b* = −0.50, *SE* = 0.11, *p* < 0.001) and concern for a friend (*b* = 0.33, *SE* = 0.04, *p* < 0.001) were significant in Step 2 (Δ*R*^2^ = 0.159). The culture and concern for a friend interaction was not significant, *b* = 0.02, *SE* = 0.07, *p* = 0.82 in Step 3 (Δ*R*^2^ = 0.0001). In sum, greater concern for a friend led people to provide more implicit support regardless of culture.

##### 4.2.5.3. Mediation analysis

**Figure 7:**
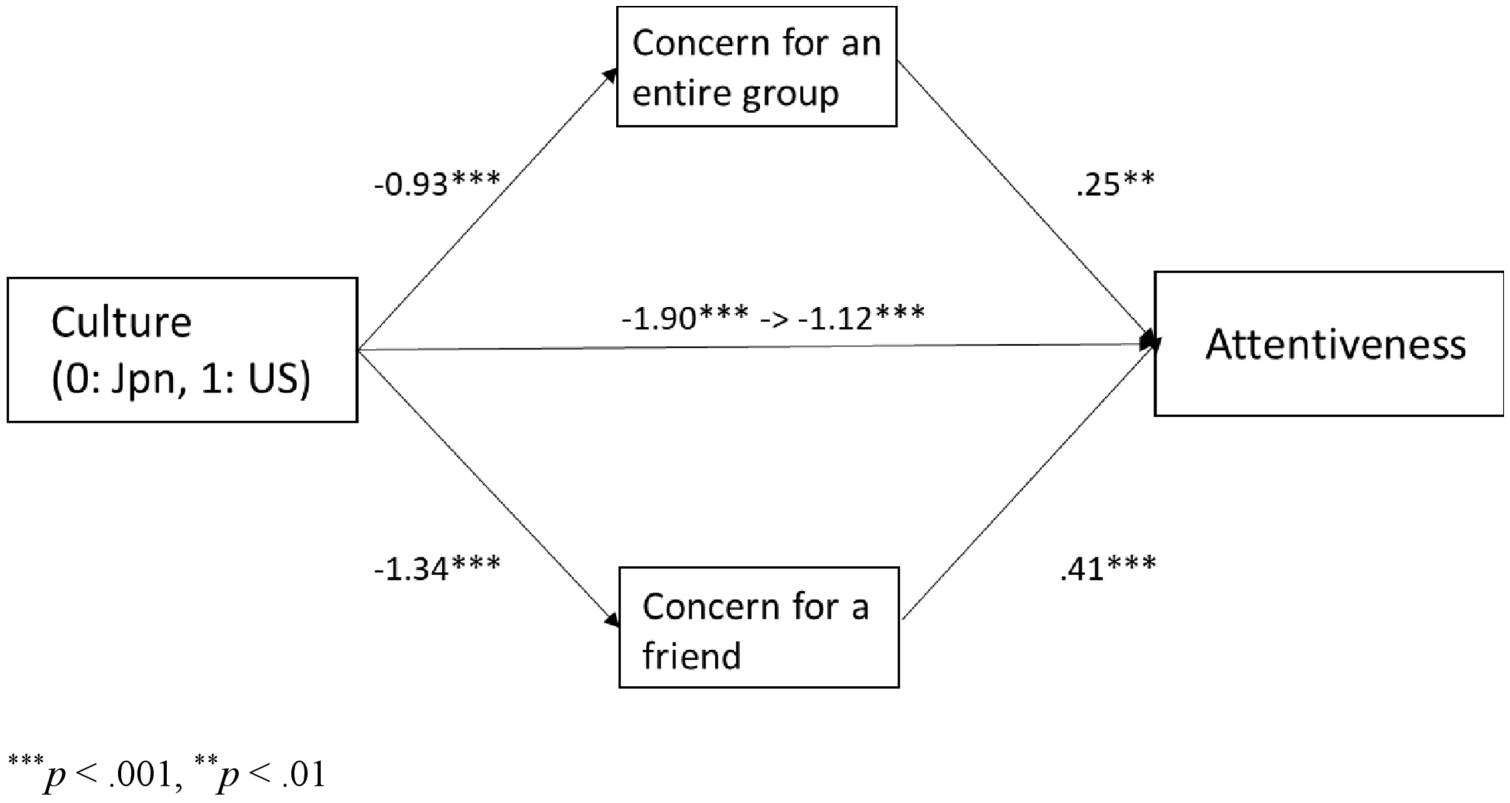
The relationship between culture and attentiveness provision, mediated by concern for an entire group, and concern for a friend in study 2.

Because the tendencies regarding relational concern motivation and implicit social support provision were similar to those in Study 1, we conducted a mediation analysis using a bootstrapping test with 2,000 replications to examine whether cultural influence on attentiveness provision was mediated by concern for an entire group motivation and concern for a friend motivation. Culture (0 = Japanese, 1 = European American) was negatively associated with attentiveness provision (*b* = −1.90, *SE* = 0.20, *p* < 0.001), concern for an entire group motivation (*b* = −0.93, *SE* = 0.11, *p* < 0.001), and concern for a friend motivation (*b* = −1.34, *SE* = 0.13, *p* < 0.001). The path was still significant when concern for an entire group motivation and concern for a friend motivation were entered as joint predictors of attentiveness provision, *b* = −1.12, *SE* = 0.25, *p* < 0.001. Concern for an entire group motivation (*b* = 0.25, *SE* = 0.10, *p* = 0.009) and concern for a friend motivation (*b* = 0.41, *SE* = 0.08, *p* < 0.001) predicted attentiveness provision (Figure 7). The indirect effect through concern for an entire group motivation (95% bias-corrected CI = [−0.43, −0.06]) and that through concern for a friend motivation (95% bias-corrected CI = [−0.80, −0.33]) were significant.

### 4.3. Discussion

Overall, the results of Study 2 replicated those of Study 1. European Americans provided more emotional and instrumental social support and had higher motivations for self-esteem and closeness compared to Japanese. Moreover, the motivations functioned as mediators: Cultural differences in emotional and instrumental social support were mediated by motivations for self-esteem and closeness that differ between the two cultures. In contrast, Japanese provided more attentiveness support and had higher concern for an entire group and concern for a friend compared to European Americans. The cultural difference in attentiveness support was mediated by the two types of relational concern motivations that differed between the cultures. Consistent with Study 1, culture moderated only the relationship between concern for an entire group motivation and implicit support provision: Higher concern for an entire group motivation increased companionship provision only in Japan, whereas it increased attentiveness provision only in the US.

## 5. General discussion

Across two studies, European Americans were more likely than Japanese to report providing emotional and instrumental support (i.e., explicit support) in response to a close other’s stressful event as well as being motivated to increase the close other’s self-esteem and the feeling of closeness. Explicit support provision was motivated by self-esteem and closeness that differ across cultures, which supports the motivation as a mediator hypothesis but not the culture as a moderator hypothesis. Reflecting the prevailing cultural values and norms regarding the view of self and interpersonal relationships, motivation for self-esteem was emphasized more in European Americans than in Japanese. It is intriguing that despite the culturally prioritized type of motivation, how the types of motivation predict the corresponding method of explicit support provision was not influenced by culture. Contrary to the findings of Chen et al. (2012), which suggested the moderating role of culture, Japanese showed significantly positive associations between motivations for self-esteem and closeness and explicit social support provision, just as European Americans did. That is, cultural differences in explicit social support provision can be attributed to variations in self-esteem and closeness motivation that differ across cultures.

In contrast, Japanese were more likely than European Americans to report providing attentiveness support in response to a close other’s stressful event, as well as having motivations for concern for an entire group and concern for a friend. Attentiveness support provision was motivated by concern for an entire group and concern for a friend that differed across cultures, which supports the motivation as a mediator hypothesis but not the culture as a moderator hypothesis. The cultural difference in attentiveness support provision can be attributed to variations in relational concerns (i.e., concern for an entire group and concern for a friend) that differ across cultures.

The validity of the findings suggesting the motivation as a mediator hypothesis should be tested in future research. The manipulation of mindsets to increase the close other’s self-esteem and the feeling of closeness, or that of mindsets to increase relational concerns, could be induced among Japanese or European Americans to examine the causal relationship between motivation and support provision. For instance, European Americans who are temporarily induced to be highly concerned about relationships by being reminded of rejection experiences may become more sensitive to potential negative impacts in the social network and the receiver by talking about stressful experiences explicitly and being provided with more implicit social support, such as attentiveness.

The current findings suggest, however, that the motivation as a mediator hypothesis does not apply, at least in part, to implicit methods of support provision. Despite the unexpected cultural difference that European Americans provided more companionship support than did Japanese, concern for an entire group motivation predicted companionship support provision only in Japanese. In contrast, concern for an entire group motivation predicted attentiveness support provision predominantly in European Americans. That is, the relationships between concern for an entire group motivation and companionship and attentiveness support provision were moderated by culture. This culture as a moderator pattern may imply two possibilities.

First, companionship support provision would be specific to Japanese providers who have a high motivation for concern for an entire group. Compared to attentiveness support provision, providers need a significant amount of time and effort to stay with the receiver. Moreover, as this research was conducted during the outbreak of COVID-19, companionship support, which requires physical and social proximity, would be particularly challenging for Japanese providers due to the collectivistic norm that promotes compliance with social distancing measures (Lu et al., 2021; Leong et al., 2022). Therefore, Japanese providers may perceive companionship support as costly. This perception might lead to the unexpected pattern of Japanese providing less companionship support than European Americans. Nevertheless, when Japanese providers feel a great level of concern and worry about the negative impacts of support provision on their social networks, they might be motivated to provide costly implicit support, such as companionship, to signal the importance of their in-groups. In this way, costly implicit support provision, such as companionship, may demonstrate the extent to which Japanese providers value their in-groups.

Second, European Americans with a strong motivation to support a group are likely to offer implicit social support, specifically attentiveness support. In fact, providing attentiveness support was not only predicted by concern for an entire group but also by concern for a friend in the US. This raises an intriguing question: Why do European Americans with high relational concerns provide attentiveness support but not companionship, even though their levels of relational concerns and attentiveness provision are relatively lower than those of the Japanese? One possible explanation is that attentiveness support may be more practical, as it allows people to be ready to provide emotional comfort and instrumental aid. Given that European Americans tend to prefer explicit social support transactions, they may believe they are expected to provide explicit support when the circumstances surrounding their friends’ situations and relationships change, and their levels of relational concerns decrease. Attentiveness may thus be placed as a preliminary step toward explicit support, particularly in European Americans with high relational concerns.

Cultural values and norms for interpersonal relationships influence the ways in which social support transactions occur and the underlying motivations behind them. For instance, motivation for self-esteem is more prominent among European Americans than among Japanese when seeking explicit support, whereas relational concern is a stronger predictor of seeking implicit support among Japanese than among European Americans (Ishii et al., 2017). The cultural differences in motivations underlying explicit and implicit support found in this research align with those in support seeking. These findings on the cultural influences on support transactions have implications for promoting smooth and cooperative interpersonal relationships in various intercultural contexts. Given the cultural differences in support transactions, even if a provider wants to uplift a partner suffering from a stressful event by boosting their self-esteem, a partner with a different cultural background may not be motivated by such an approach. On the other hand, even if a provider believes that being attentive without self-disclosure is a good strategy to avoid further distressing a partner going through a stressful event, it may not align with the expectations of a partner with a different cultural background. Knowledge about the cultural impact on support transactions, which this present research contributes to, is crucial to prevent miscommunication between support providers and seekers.

This research provides additional evidence of cultural and motivational influences on implicit social support provision. To investigate these influences, we proposed two types of motivation related to implicit social support provision: concern for an entire group and concern for a friend. We also focused on two types of implicit support: companionship and attentiveness. The proposed distinction regarding relational concerns contributes to a better understanding of the culturally specific effects on the association between relational concerns and implicit social support provision. Concern for a friend predicted attentiveness support provision regardless of culture, suggesting that it would likely cause individuals to hold back explicit support provision that might strain a friend’s feelings and reputation. Instead, they would choose to provide support in a more implicit but less costly way. In contrast, the effect of concern for an entire group was culturally specific. Specifically, its association with companionship in Japan was in line with previous work, suggesting that the assumed association between relational concerns and implicit social support would mainly refer to concern for a group, which is not limited to the recipient themselves, and companionship, which is a relatively costly form and likely signals an individual’s consideration for the in-group. Given the nature of concern for an entire group and companionship, the necessity for individuals to have such a concern and provide such a type of support would be greater in a culture that mandates maintaining harmonious interpersonal relationships.

The present research raises some issues that need to be addressed in future studies. First, because we asked participants to recall stressful events that happened to themselves (Study 1) or their friends (Study 2), it is unclear to what extent their retrospective responses to their ways of support provision and underlying motivation accurately reflected their genuine psychological and behavioral tendencies. To reach more valid conclusions, future research needs to measure participants’ behavioral responses to their friends’ stressful events in a laboratory setting—for instance, how they handle a situation when a co-participating friend struggles with an experimental task. Second, this research relied on a cross-sectional design, so the role of motivation as a mediator remains unclear. Given the first limitation regarding participants’ retrospective responses, they might rate the items of motivation to justify their response to support provision. If so, support provision methods might predict the corresponding types of motivation instead. Third, we did not control the types of stressful events. It cannot be ruled out that cultural differences might result from a specific type of stressful event that was reported disproportionately across cultures. This issue should be addressed in a more controlled manner, such as in a laboratory setting, in future work. Finally, although we controlled for the potential influence of the friend’s instrumentality, which was higher in European Americans than in Japanese in both studies, we did not address how the concrete features of friendships (e.g., the length of the friendship and the origin of the friendship) influence social support prevision and the underlying motivations. Indeed, the cultural difference in the mean score of friend’s instrumentality implies that the representation of the “closest friend” might vary between Japanese and European Americans. Additionally, the concrete features of friendships would differ across cultures due to socioecological factors such as residential mobility (Oishi, 2010) and relational mobility (Schug et al., 2010). Showing one’s goodness and trustworthiness through emotional and instrumental support would be more important in a mobile environment where interpersonal relationships consist of relative weak ties compared to a stable environment where interpersonal relationships are fixed. Therefore, future research should further address the motivations of providers and the corresponding ways of support provision.

Despite these limitations, our findings regarding the motivational underpinnings of explicit and implicit support provision across cultures will contribute to our understanding of social support provision within the context of cultural practices and norms. Our research underscores the role of motivation, which differs across cultures and has not been fully addressed. Our results also suggest that certain aspects of relational concerns are culturally driven and, accordingly, lead to culturally specific patterns of implicit support provision, which poses further questions for investigation in future work.

## Data availability statement

The raw data supporting the conclusions of this article will be made available by the authors, without undue reservation.

## Ethics statement

The studies involving human participants were reviewed and approved by Nagoya University, Graduate School of Informatics, Department of Cognitive, and Psychological Sciences. The patients/participants provided their written informed consent to participate in this study.

## Author contributions

RT and KI conceptualized and designed the research, prepared the materials, and collected and analyzed data. RT wrote the first draft. SZ and KI edited and finalized the manuscript. All authors contributed to the article and approved the submitted version.

## Funding

This work was supported by JSPS KAKENHI Grant Number 19 K21812.

## Conflict of interest

The authors declare that the research was conducted in the absence of any commercial or financial relationships that could be construed as a potential conflict of interest.

## Publisher’s note

All claims expressed in this article are solely those of the authors and do not necessarily represent those of their affiliated organizations, or those of the publisher, the editors and the reviewers. Any product that may be evaluated in this article, or claim that may be made by its manufacturer, is not guaranteed or endorsed by the publisher.

## Footnotes

^1^Chen et al. (2015) found that relationship quality was positively associated with emotion-focused support provision, particularly in European Americans, whereas it was positively associated with implicit support provision, especially among Asian Americans. Based on the finding, we performed an ANCOVA by adding the interaction term of culture and the friend’s instrumentality to these covariates. However, neither the culture and the friend’s instrumentality interaction (*F*(1, 427) = 0.44, *p* = 0.51) nor the interaction including support (*F*(3, 1,281) = 0.96, *p* = 0.41) were significant.

^2^As in Study 1, we performed an ANCOVA by adding the interaction term of culture and the friend’s instrumentality to these covariates. However, neither the culture and the friend’s instrumentality interaction (*F*(1, 574) = 3.68, *p* = 0.056) nor the interaction including support (*F*(3, 1722) = 1.82, *p* = 0.14) were significant.
