# Supplementary material for: Cultural differences in explicit and implicit support provision and underlying motivations for self-esteem, closeness, and relational concerns
Source: Front Psychol. 2023 Aug 3;14:1202729. doi: 10.3389/fpsyg.2023.1202729 (PMC10435086; doi:10.3389/fpsyg.2023.1202729)
Supplement: Supplementary file 1 [file Data_Sheet_1.pdf]

Cultural Differences in Explicit and Implicit Support Provision and Underlying Motivations for Self-esteem, Closeness, and Relational Concerns

Supplemental Materials

Study 1

An Exploratory Factor Analysis for Support Provision Items

We performed an exploratory factor analysis of the 12 support provision items, utilizing the generalized least squares method with Promax rotation separately for each culture. The analysis with a cut-off point of .40 yielded a four-factor solution as the best fit for the data in both cultures, accounting for a total of 56% of the variance among European Americans and 60% of the variance among Japanese. Factor 1 explained 32% of the variance among European Americans and 33% of the variance among Japanese and was labeled "instrumental support provision". Factor 2 explained an additional 32% of the variance among European Americans and an additional 29% of the variance among Japanese and was labeled "companionship". Factor 3 explained an additional 19% of the variance among European Americans and an additional 20% of the variance among Japanese and was labeled "emotional support provision". And factor 4 explained an additional 18% of the variance among European Americans and an additional 17% of the variance among Japanese and was labeled "attentiveness." These results suggest that the four different types of social support are commonly found among both European Americans and Japanese (Table S1).

An Exploratory Factor Analysis for Motivation Items

We performed an exploratory factor analysis of the 28 motivation items, utilizing the principal component method with Promax rotation separately for each culture. Using a cut-off point of .40, the analysis produced a six-factor solution that best fit the data in both cultures,

although the items comprising some of the factors differed between the two cultures. Therefore, we selected 20 items that were consistently included in the factors across cultures and conducted an exploratory factor analysis on these items, utilizing the principal component method with Promax rotation separately for each culture. Once again using a cut-off point of .40, the analysis yielded a six-factor solution that provided the best fit for the data in both cultures, accounting for a total of 57.3% of the variance among European Americans and 60.3% of the variance among Japanese. Factor loadings for each item among European Americans and Japanese are presented in Tables S2 and S3, respectively. In European Americans, factor 1 explained 17.9% of the variance and was labeled “concern for an entire group”. Factor 2 explained an additional 17.0% of the variance and was labeled “concern for a friend”. Factor 3 explained an additional 17.3% of the variance and was labeled “low efficacy”. Factor 4 explained an additional 19.0% of the variance and was labeled “self-esteem”. Factor 5 explained an additional 17.9% of the variance and was labeled “closeness”. Factor 6 explained an additional 10.8% of the variance and was labeled “self-improvement”. In Japanese, factor 1 explained 20.2% of the variance and was labeled “concern for a friend”. Factor 2 explained an additional 17.5% of the variance and was labeled “low efficacy”. Factor 3 explained an additional 16.9% of the variance and was labeled “self-esteem”. Factor 4 explained an additional 16.7% of the variance and was labeled “closeness”. Factor 5 explained an additional 16.3% of the variance and was labeled “concern for an entire group”. Factor 6 explained an additional 12.5% of the variance and was labeled “self-improvement”.

Relationship between Motivation and Implicit Support Provision: Self-Improvement and Low Efficacy

We also analyzed relationships between each of the two types of implicit support (companionship and attentiveness) and self-improvement and low efficacy.

Self-Improvement

For each implicit support, culture and self-improvement were entered along with the control variables of the negative and solvable ratings for stressors, the friend's instrumentality, gender, and age (Step 1). The interaction between culture and self-improvement was then added (Step 2). For companionship, the main effect of self-improvement was significant, $b = .21$, $SE = 0.05$, $p < .001$ in Step 1 ($R^2 = .169$). The culture and self-improvement interaction was not significant, $b = .01$, $SE = 0.11$, $p = .95$ in Step 2 ($\Delta R^2 = .000$). For attentiveness, the main effect of self-improvement was significant, $b = .36$, $SE = 0.04$, $p < .001$ in Step 1 ($R^2 = .221$). The culture and self-improvement interaction was not significant, $b = .15$, $SE = 0.09$, $p = .09$ in Step 3 ($\Delta R^2 = .005$). In sum, greater motivation for self-improvement led people to provide more implicit support regardless of culture.

Low Efficacy

For each implicit support, culture and low efficacy were entered along with the control variables of the negative and solvable ratings for stressors, the friend's instrumentality, gender, and age (Step 1). The interaction between culture and low efficacy was then added (Step 2). For companionship, although the main effect of low efficacy was not significant, $b = .08$, $SE = 0.05$, $p = .08$ in Step 1 ($R^2 = .144$), the culture and low efficacy interaction was significant, $b = -.26$, $SE = 0.09$, $p = .004$ in Step 2 ($\Delta R^2 = .017$). Lower efficacy led Japanese to provide more companionship ($b = .20$, $SE = 0.06$, $p = .001$), whereas the tendency disappeared in European Americans ($b = -.06$, $SE = 0.07$, $p = .38$). For attentiveness, the main effect of low efficacy was

significant, *b* = .19, *SE* = 0.04, $p < .001$ in Step 1 (R^2 = .142). The culture and self-improvement interaction was not significant, *b* = .06, *SE* = 0.08, *p* = .48 in Step 3 (ΔR^2 = .001).

Study 2

A Confirmation Factor Analysis for Support Provision Items

To confirm the four-factor hierarchical model of support provision items suggested by Study 1, we conducted a confirmatory factor analysis of the 12 items for each culture. We used maximum likelihood extraction to estimate the model. The model fit was acceptable for each culture ($\chi^2(48) = 107.66$, $p < .001$, CFI = .953, RMSEA = .066 for European Americans [see Figure S1], and $\chi^2(48) = 234.50$, $p < .001$, CFI = .880, RMSEA = .115 for Japanese [see Figure S2]). Therefore, we adopted this model for the successive testing. The labels of factors were identical to those used in Study 1.

A Confirmation Factor Analysis for Motivation Items

To confirm the six-factor hierarchical model of motivation items suggested by Study 1, we conducted a confirmatory factor analysis of the 20 items for each culture. We estimated the model for each culture using maximum likelihood extraction. Although the model fit was acceptable for each culture, one item related to self-improvement had a low factor loading in both cultures. To address this, we excluded the item and conducted a confirmatory factor analysis for each culture again ($\chi^2(137) = 260.18$, $p < .001$, CFI = .946, RMSEA = .056 for European Americans [see Figure S3], and $\chi^2(137) = 342.09$, p < .001, CFI = .908, RMSEA = .072 for Japanese [see Figure S4]). We adopted this revised model for the successive testing, with factor labels identical to those used in Study 1.

Relationship between Motivation and Implicit Support Provision: Self-Improvement and Low Efficacy

As in Study 1, we also analyzed relationships between each of the two types of implicit support (companionship and attentiveness) and self-improvement and low efficacy.

Self-Improvement

For each implicit support, culture and self-improvement were entered along with the control variables of stressful rating for stressors, the friend's instrumentality, gender, and age (Step 1). The interaction between culture and self-improvement was then added (Step 2). For companionship, the main effect of self-improvement was significant, b = .22, SE = 0.04, p < .001 in Step 1 (R^2 = .130). The culture and self-improvement interaction was not significant, b = -.09, SE = 0.08, p = .27 in Step 2 (ΔR^2 = .002). For attentiveness, the main effect of self-improvement was significant, b = .25, SE = 0.03, p < .001 in Step 1 (R^2 = .228). The culture and self-improvement interaction was not significant, b = .09, SE = 0.07, p = .21 in Step 3 (ΔR^2 = .002). In sum, greater motivation for self-improvement led people to provide more implicit support regardless of culture.

Low Efficacy

For each implicit support, culture and low efficacy were entered along with the control variables of the stressful rating for stressors, the friend's instrumentality, gender, and age (Step 1). The interaction between culture and low efficacy was then added (Step 2). For companionship, neither the main effect of low efficacy (b = .06, SE = 0.04, p = .21) in Step 1 (R^2 = .089) nor the culture and low efficacy interaction (b = -.08, SE = 0.09, p = .40 in Step 2 (ΔR^2 = .001) was significant. For attentiveness, the main effect of low efficacy was significant, b = .18, SE = 0.04, p < .001 in Step 1 (R^2 = .190). The culture and self-improvement interaction was significant, b = .28, SE = 0.07, p < .001 in Step 3 (ΔR^2 = .020). Lower efficacy led European

Americans to provide more attentiveness (b = .30, SE = 0.05, p < .001), whereas the tendency disappeared in Japanese (b = .02, SE = 0.05, p = .71).

Table S1

Factor Loadings for Support Provision Items for Each Culture in Study 1

Items	Factor 1	Factor 2	Factor 3	Factor 4
I would give the friend some advice that will be useful in dealing with the problem. (I1)	**.97**<br>**.92**	.04<br>-.12	-.05<br>.00	-.08<br>.02
I would suggest how to solve the problem to the friend. (I2)	**.92**<br>**.91**	.03<br>-.07	-.18<br>-.05	-.02<br>-.05
I would help the friend to think about the problem more clearly. (I3)	**.55**<br>**.80**	.10<br>.11	.17<br>.04	.01<br>-.01
I would increase the time I spend with the friend without talking about his/her problem. (C1)	-.08<br>-.10	**.84**<br>**.92**	.16<br>-.02	-.07<br>-.07
I would increase the time I spend with the friend, even without talking. (C2)	.14<br>.09	**.78**<br>**.85**	.09<br>.07	-.14<br>-.11
I would hang out with the friend without talking about the cause of his/her stress. (C3)	.05<br>-.12	**.74**<br>**.68**	-.19<br>-.12	.21<br>.13
I would offer the friend words of comfort, such as "I know you've had a tough time." (E1)	-.10<br>-.05	-.01<br>-.12	**.85**<br>**.83**	-.08<br>.05
I would encourage the friend by saying things like, "Don't worry, it's going to be all right." (E2)	.01<br>-.01	.23<br>.17	**.50**<br>**.55**	.00<br>.07
I would tell the friend how important he/she is to me. (E3)	.29<br>.07	-.14<br>-.02	**.32**<br>**.68**	.14<br>-.11
I would just wait until the friend asked me for advice, but in the meantime, I would keep caring about him/her and his/her situation. (A1)	.05<br>-.11	-.17<br>-.04	-.02<br>.05	**.75**<br>**.72**
I would keep a little distance until the friend felt better although I cared if he/she is okay. (A2)	-.10<br>.01	.23<br>-.06	-.14<br>-.05	**.56**<br>**.75**
I would care for the friend so that I could always support him/her although I would leave him/her alone until he/she could clear his/her mind. (A3)	.01<br>.20	.05<br>.24	.24<br>.03	**.49**<br>**.35**

Note. For each item, upper numbers present factor loadings in European Americans, while lower numbers present factor loadings in Japanese.

Table S2

Factor Loadings for Motivation for Providing Support Items among European Americans in Study 1

Items	Factor 1	Factor 2	Factor 3	Factor 4	Factor 5	Factor 6
I don't want to disrupt the harmony of a group by talking to the depressed friend. (CG1)	**.79**	.04	-.06	-.07	.10	-.02
If I let the friend disclose the problem to me, I would feel embarrassed. (CG2)	**.73**	-.09	.06	-.05	.05	.03
I should not ask about the friend’s problem in order to maintain happiness among my group of friends. (CG3)	**.59**	.13	-.04	.01	.05	.04
I don't want to get involved in the friend's problem. (CG4)	**.45**	-.05	.32	.11	-.11	-.12
I don't want to give the friend any stress by asking about the problem he/she is having. (CF1)	.07	**.86**	.00	-.02	-.06	-.25
I don't want to hurt the friend more by asking about a problem he/she is having. (CF2)	-.02	**.75**	.00	.03	-.03	.01
I don't want to bother the friend any further by making it a big deal. (CF3)	.00	**.62**	.04	-.16	.09	.24
I don't want to make the friend's problem bigger. (CF4)	-.02	**.46**	.01	.33	-.08	.11
I'm not good enough to help the friend deal with the problem. (L1)	-.08	-.03	**.89**	.13	-.08	.01
I don't think there's anything I can do for the friend. (L2)	-.04	.06	**.76**	-.17	.10	.08
I don't know how to react to the friend who is having trouble. (L3)	.24	.03	**.58**	.10	-.03	-.03
I want the friend to be able to feel good about him/herself. (SE1)	.00	.01	-.01	**.77**	.13	-.11

I want the friend to be confident in him/herself. (SE2)	-.06	-.12	.11	**.73**	.12	.24
I want the friend to have high self-esteem. (SE3)	.00	.06	-.01	**.66**	.14	.02
I want the friend to feel close to me. (C1)	.19	-.08	.03	.08	**.91**	-.07
I want the friend and I to be able to feel close to each other. (C2)	.11	-.09	-.09	.16	**.78**	.00
I want the friend to know that I do care about him/her. (C3)	-.28	.15	.02	.18	**.52**	-.08
I believe that the friend can overcome the problem on his/her own. (SI1)	-.11	-.09	.08	.06	-.04	**.74**
I want the friend to work on his/her own and develop. (SI2)	.27	.01	-.21	.19	-.13	**.53**
I want to let the friend struggle in his/her own way. (SI3)	.14	.05	.12	-.13	.07	**.37**

Table S3

Factor Loadings for Motivation for Providing Support Items among Japanese in Study 1

Items	Factor 1	Factor 2	Factor 3	Factor 4	Factor 5	Factor 6
I don't want to give the friend any stress by asking about the problem he/she is having. (CF1)	**.92**	.00	-.17	.04	-.07	-.08
I don't want to bother the friend any further by making it a big deal. (CF3)	**.80**	-.07	-.01	-.10	.10	.00
I don't want to hurt the friend more by asking about a problem he/she is having. (CF2)	**.78**	.08	-.06	.10	-.02	.06
I don't want to make the friend's problem bigger. (CF4)	**.53**	.04	.26	-.05	.05	.03
I'm not good enough to help the friend deal with the problem. (L1)	.06	**.97**	.09	.02	-.21	-.01
I don't know how to react to the friend who is having trouble. (L3)	.05	**.74**	.00	.07	.10	-.01
I don't think there's anything I can do for the friend. (L2)	-.08	**.72**	-.08	-.03	.16	-.04
I want the friend to have high self-esteem. (SE3)	-.14	.01	**.81**	.01	.18	-.05
I want the friend to be able to feel good about him/herself. (SE1)	-.08	.10	**.74**	.06	-.07	.12
I want the friend to be confident in him/herself. (SE2)	.17	-.12	**.71**	.00	-.05	.02
I want the friend to feel close to me. (C1)	.01	-.03	-.19	**.96**	.06	.08
I want the friend and I to be able to feel close to each other. (C2)	.04	.05	.14	**.70**	.01	-.03
I want the friend to know that I do care about him/her. (C3)	-.04	.02	.15	**.68**	.01	-.03
I don't want to disrupt the harmony of a group by talking to the depressed friend. (CG1)	.02	-.09	-.03	.17	**.78**	.00

I should not ask about the friend's problem in order to maintain happiness among my group of friends. (CG3)	.07	.04	.18	-.04	**.68**	-.09
If I let the friend disclose the problem to me, I would feel embarrassed. (CG2)	-.04	-.04	.05	.01	**.68**	.02
I don't want to get involved in the friend's problem. (CG4)	.00	.18	-.19	-.18	**.52**	.11
I want the friend to work on his/her own and develop. (SI2)	-.05	-.09	-.04	.02	-.02	**.92**
I want to let the friend struggle in his/her own way. (SI3)	.02	.04	-.05	.05	.07	**.55**
I believe that the friend can overcome the problem on his/her own. (SI1)	.01	.01	.18	-.06	-.05	**.54**

Figure S1

Construct Validity of the Four-Factor Model of Support Provision Items with Standardized Coefficients in European Americans in Study 2

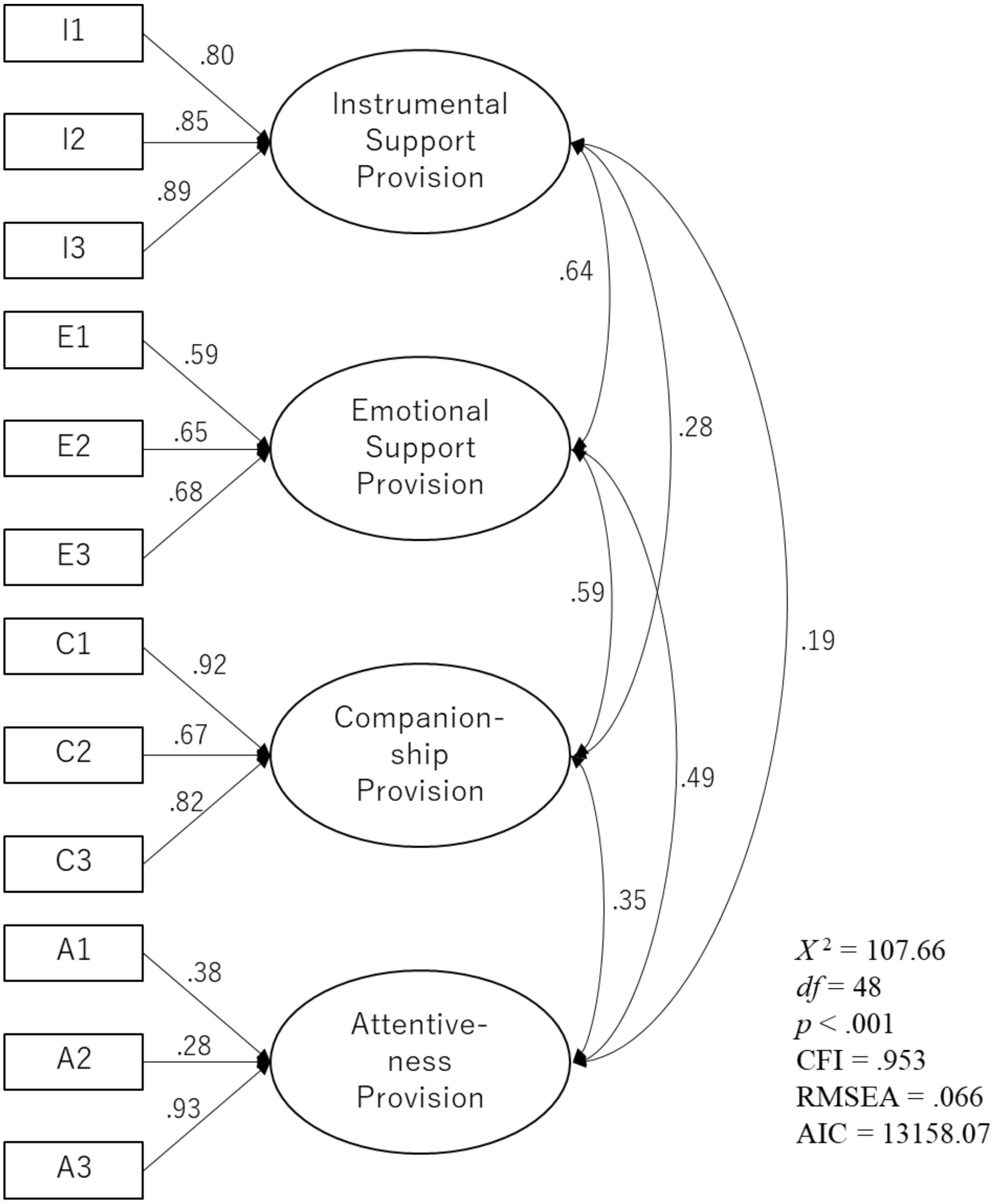

Note. Items were named in Tables S1 and S2. Insignificant covariances were not presented.

Figure S2

Construct Validity of the Four-Factor Model of Support Provision Items with Standardized Coefficients in Japanese in Study 2

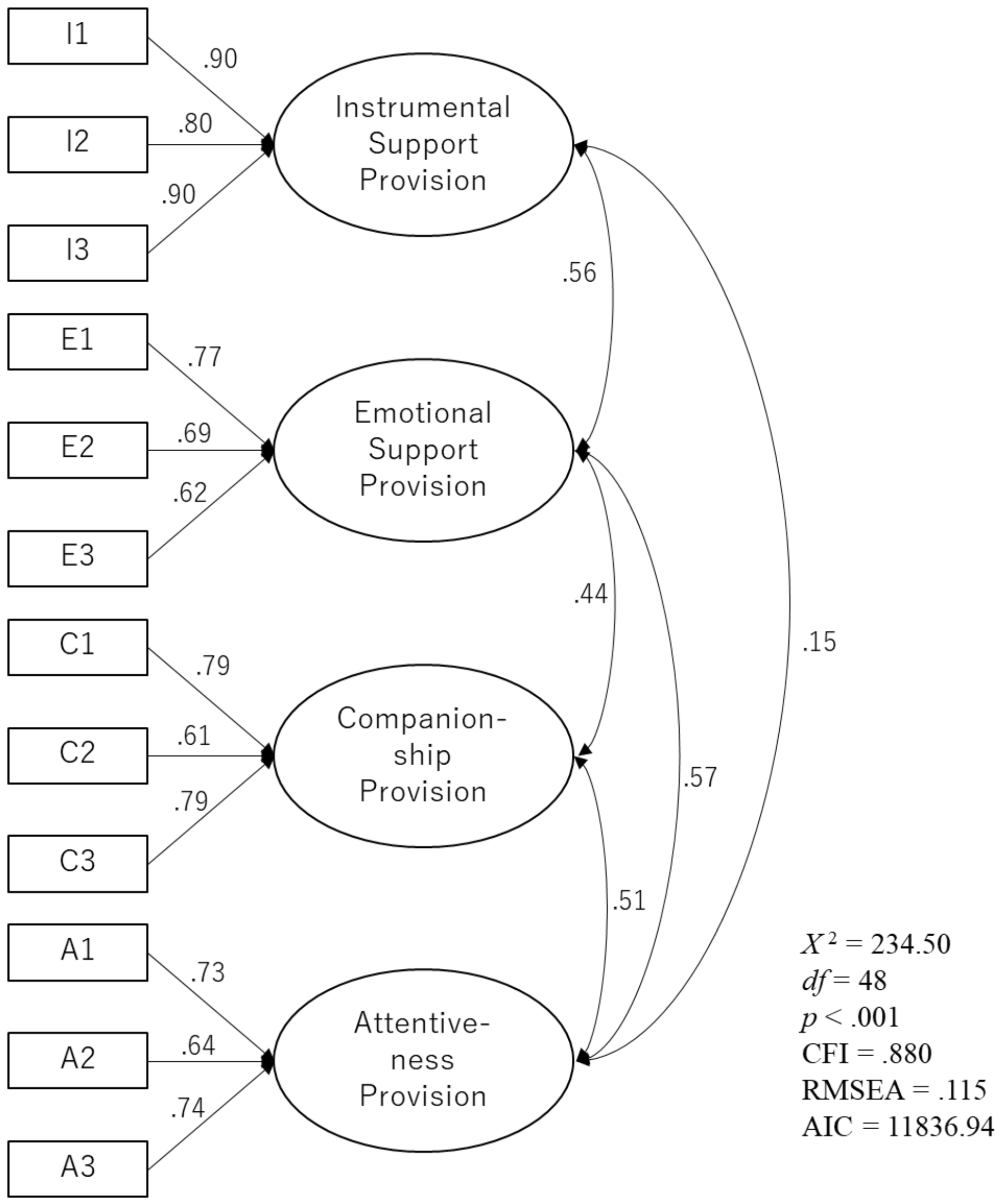

Note. Items were named in Tables S1 and S2. Insignificant covariances were not presented.

Figure S3

Construct Validity of the Six-Factor Model of Motivation Items with Standardized Coefficients in European Americans in Study 2

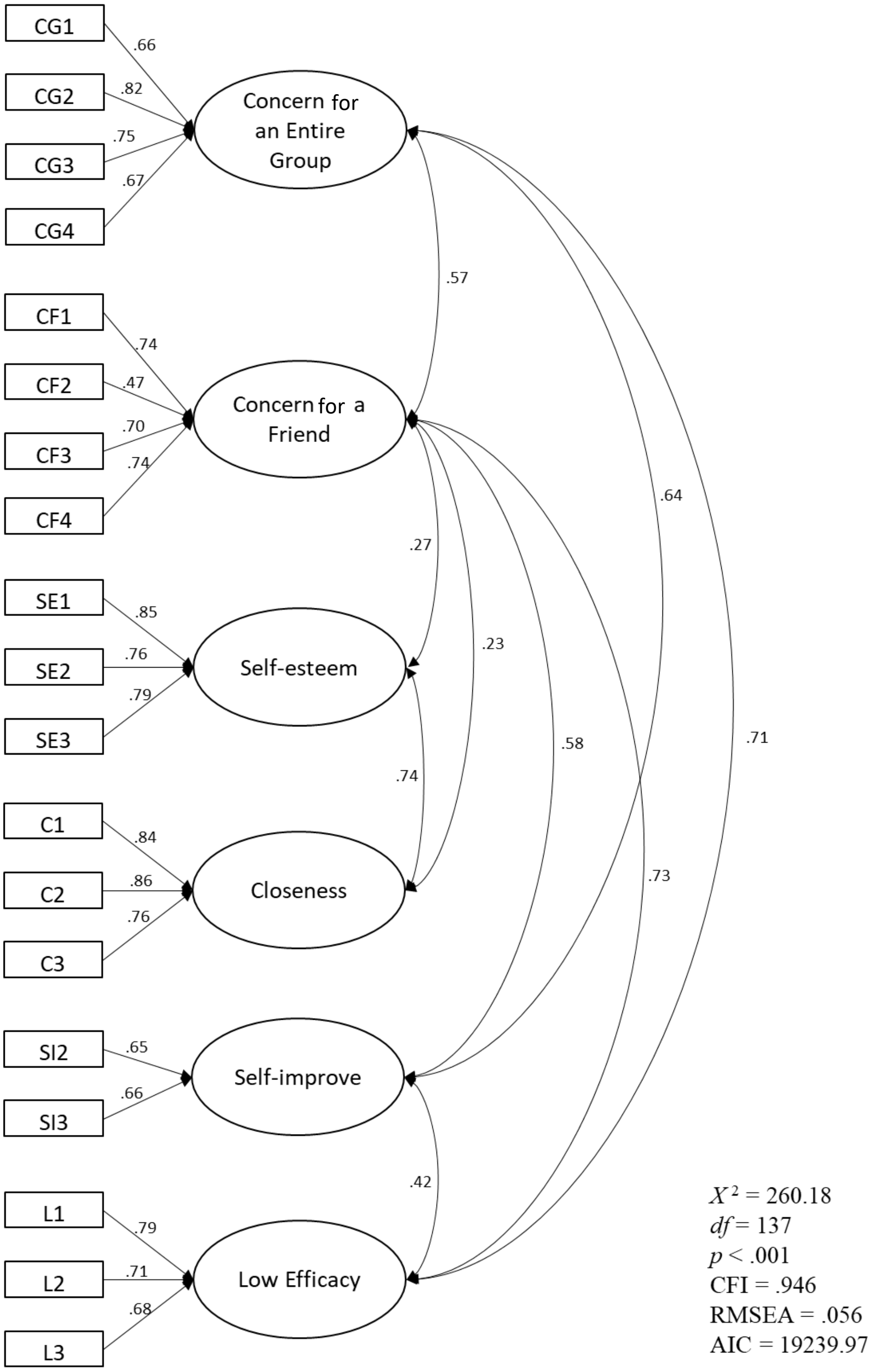

Note. Items were named in Tables S3 and S4. Insignificant covariances were not presented.

Figure S4

Construct Validity of the Six-Factor Model of Motivation Items with Standardized Coefficients in Japanese in Study 2

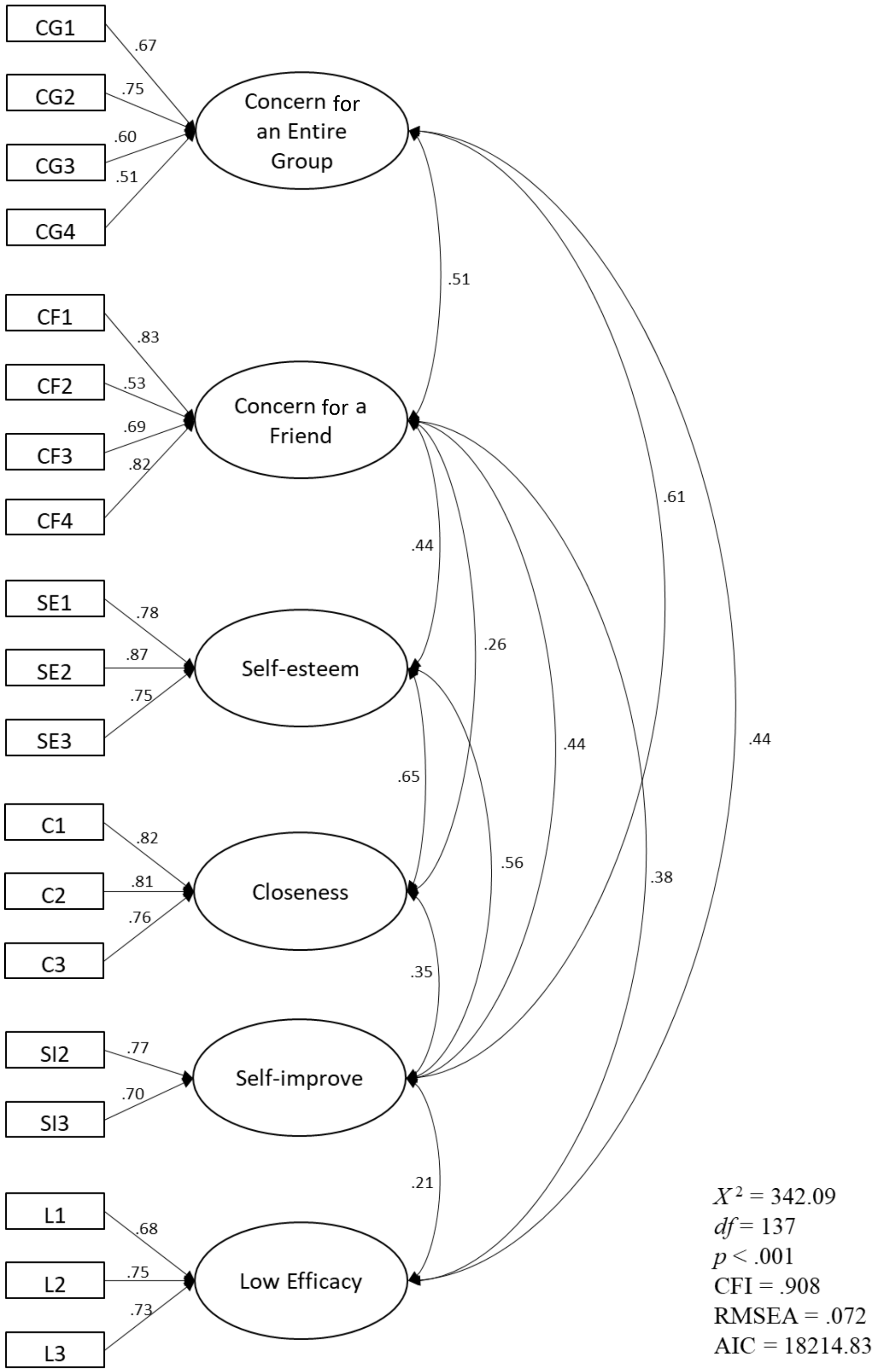

Note. Items were named in Tables S3 and S4. Insignificant covariances were not presented.
